# Application of Modulators of Ca^2+^-Activated Big-Conductance Potassium Channels Against Cd^2+^-Induced Cytotoxicity: A Study on Two Rat Cell Lines, PC12 and AS-30D

**DOI:** 10.3390/ijms262010048

**Published:** 2025-10-15

**Authors:** Elena A. Belyaeva, Tatyana V. Sokolova

**Affiliations:** Sechenov Institute of Evolutionary Physiology and Biochemistry, Russian Academy of Sciences, 44 Thorez Avenue, Saint-Petersburg 194223, Russia

**Keywords:** Cd^2+^-induced mitochondrial dysfunction-mediated cytotoxicity, NS004, NS1619, paxilline, Ca^2+^-activated big-conductance potassium channels, mitochondrial respiratory chain, reactive oxygen species, PC12 pheochromocytoma, AS-30D ascites hepatoma

## Abstract

As we found earlier, paxilline (a Penicillium paxilli mycotoxin and blocker of Ca^2+^-activated big-conductance potassium channels, BK(Ca)s) attenuated Cd^2+^-induced cytotoxic effects, whereas BK(Ca) activators (NS004, NS1619) and Cd^2+^ were able to induce apoptosis, which was enhanced when used together. In this work, molecular mechanisms underlying the aforementioned effects were studied using two rat cell lines, PC12 and AS-30D, flow cytometry, and spectrofluorometric and polarographic techniques. Both NS004 and NS1619 were found to have time- and dose-dependent effects on cell viability, respiration, mitochondrial membrane potential, and intracellular reactive oxygen species (ROS) production. In PC12 cells, BK(Ca) openers exerted an uncoupling effect after 3 h, increasing the resting respiration, while they partially inhibited the maximal respiration after 5 and 24 h; in addition, after 3 h, a transient protection by NS004/NS1619 against Cd^2+^-induced decrease of cell viability was observed. In both cell types, NS004/NS1619 increased ROS production after 3 h and counteracted the mitigating effect of paxilline against Cd^2+^-induced necrosis. In turn, paxilline reduced NS004/NS1619-induced apoptosis in AS-30D cells and ROS increase produced by NS004/NS1619 and/or Cd^2+^ in PC12 cells. As a result, the involvement of the mitochondrial respiratory chain, ROS, and, very likely, BK(Ca)s, in the mechanisms of the modulatory effects of the BK(Ca) blocker/opener(s) used in the absence and presence of Cd^2+^ was revealed.

## 1. Introduction

Metal/metalloid contamination, as a part of the growing environmental pollution, has become a serious problem in recent decades. Exposure to metal/metalloid(s) from the environment is dangerous for all living beings, although to different degrees [1,2,3,4]. Nowadays, heavy metals are considered as one of the main stressors entering the environment, from which they are absorbed by humans and animals and turn out to be the major source of various diseases and pathological conditions [5,6,7,8,9,10,11]. It is known that the brain, liver, kidney, lungs, endocrine system, and many other organs and tissues, and especially their mitochondria, are critical targets for heavy metals [12,13,14,15,16,17,18,19,20,21,22,23]. In this regard, an elucidation of molecular mechanisms of toxicity/tolerance to heavy metals, particularly the mechanisms of interference of these environmental contaminants/xenobiotics in cellular processes, as well as a search for possible interventions to block or enhance the effects of heavy metals, is vital.

Cadmium (Cd^2+^) is one of the most harmful carcinogenic and cyto- and genotoxic heavy metal ions with a half-life of several decades [1]. The most prominent characteristic of this metal is its action with high affinity not only on dithiol binding sites but also on Ca^2+^ binding sites (of note, the crystal ionic radius of Cd^2+^ (0.097 nm) is close to that for Ca^2+^ (0.099 nm)) [1,12,13,14,23,24]. In addition, some time ago we revealed the discrete modes of Cd^2+^ effect on calcium- and thiol-dependent membrane domains crucial for mitochondrial function; namely, this divalent heavy metal ion can induce the increased membrane permeabilization of rat liver mitochondria (RLM) through Ca^2+^-dependent domain independently of its effects on the critical dithiols [25,26,27]. Usage of heavy metal ions (Cd^2+^, Hg^2+^, Cu^2+^ etc.), not only as research objects but also as tools to study the functioning of various cellular/organellar channels/structures, such as (i) mitochondrial permeability transition pores, mPTP [28,29] (see, for example, our papers [13,14,25,27,30,31,32]), (ii) selective potassium channels [28], namely ATP-sensitive potassium channels, K(ATP) [33,34] and BK(Ca) [35,36,37], (iii) mitochondrial electron transport chain, mETC [14,27,38,39,40,41,42,43,44,45,46] and many others, as well as for elucidating the role of these channels/structures in the regulation of essential cellular processes (ion and redox homeostasis, energy metabolism, cell signaling and aging, cell death and cytoprotective responses of various types) is extremely important.

The large-conductance calcium- and voltage-activated potassium channel (BK(Ca), or MaxiK, or K_Ca_1.1, or Slo1) is a transmembrane protein with a high conductance and selectivity for K^+^, which is encoded by the *KCNMA1* gene and is widely distributed in excitable cells (central nervous system, muscle, etc.); however, it is also found in non-excitable tissues such as bone, salivary, kidney, liver, and many others [36,37,47]. As generally accepted, this channel regulates excitability, Ca^2+^ signaling, and K^+^ efflux. Notably, BK(Ca) resides not only in the plasma membrane (pBK(Ca)) but also in mitochondrial (mBK(Ca)) and nuclear (nBK(Ca)) membranes, and these channels share most of the biophysical and pharmacological properties with pBK(Ca) [28,47]. mBK(Ca) is found in the inner mitochondrial membrane (IMM) of different cells and tissues, namely astrocytes, neurons, myocytes, liver and kidney cells, various endothelial cells, and many others, including different types of cancer cells. Despite the fact that the mBK(Ca) is also encoded by the *KCNMA1* gene, it is a splice variant containing a C-terminal DEC splice insert that determines mitochondrial targeting (refs. [28,47] and references therein).

As we have previously found in a series of in vitro experiments using several BK(Ca) modulators, they influence the toxic effects of Cd^2+^ on mitochondrial and cellular function. In particular, paxilline (Pax), a lipophilic mycotoxin and non-peptide alkaloid from the fungus Penicillium paxilli and a potent BK(Ca) inhibitor, partially protects against Cd^2+^-induced cytotoxicity [35,48], whereas BK(Ca) openers, namely NS004 (5-trifluoromethyl-1-(5-chloro-2-hydroxyphenyl)-1,3-dihydro-2H-benzimidazole-2-one) and NS1619 (1,3-dihydro-1- [2-hydroxy-5-(trifluoromethyl) phenyl]-5-(trifluoromethyl)-2H-benzimidazole-2-one), i.e., its synthetic agonists, enlarge the apoptosis-inducing effect of Cd^2+^ [49]. As recognized, an application of modulators of various ion channels to study molecular mechanisms of possible contribution of these channels in different pathological conditions/diseases and cell destiny is very fruitful. As to mBK(Ca), its activation is generally considered to be cardio- and neuroprotective [28,36,37,47,50]; however, BK(Ca) activators can also induce/enhance cell death of different types, especially in malignant cells [49,51,52,53,54,55]. At present, both BKCa agonists and antagonists have been shown to be anticancer agents [56]. It should be reminded that NS004 was the first BK(Ca) activator discovered; subsequently, NS1619 became one of the most often applied synthetic openers of BK(Ca) in both in vivo and in vitro experiments. Furthermore, nowadays, the benzimidazoles or benzimidazole derivatives (NS004, NS1619, NS11021, and so on) are indispensable in such kinds of experiments and widely used as activators of both cellular and organellar BK(Ca)s, in spite of the fact that they have a number of “side effects” (or additional cellular targets) [28,47,50,56,57]. As to Pax (a tremorgenic indole-diterpene), it is one of the most powerful and selective BK(Ca) blockers, which is extensively used in different types of experiments because of its apparent high specificity and reversibility [36]. Nevertheless, to date, additional cell targets for this BK(Ca) antagonist have been found as well (refs. [35,47,57] and references therein). Notably, Pax has been shown to be cytoprotective in some cases, but the mechanisms of its improving action are not completely clear [35,48,49,58].

The objective of this study was to underscore the mechanisms underlying the relationship between the effects of Cd^2+^, NS004/NS1619, and Pax and to clarify the possible role of BK(Ca)s in these events. For this purpose, we used two types of rat cell lines to continue the study of the molecular mechanisms of Cd^2+^-induced neuro- and hepatotoxicity, namely pheochromocytoma PC12 and ascites hepatoma AS-30D, which we applied in our previous works on the issue [35,49], as well as flow cytometry, polarographic, and spectrofluorometric methods. Overall, it has been established that mETC, ROS, and, highly likely, BK(Ca)s, are the key players involved in the modulation of Cd^2+^ cytotoxicity by the BK(Ca) inhibitor/activator(s). A portion of this study has been previously published in the form of abstracts [59,60].

## 2. Results

### 2.1. Action of BK(Ca) Modulators on PC12 Cells in the Absence and in the Presence of Cd^2+^

At first, we studied the action of BK(Ca) activators, namely NS004 and NS1619, on the functioning of PC12 cells; in particular, we measured their effects on cell viability, respiration, mitochondrial membrane potential (ΔΨ_mito_), and intracellular ROS production in the absence and in the presence of Cd^2+^ and/or Pax.

#### 2.1.1. Effects of BK(Ca) Modulators on Viability of PC12 Cells

**Figure 1:**
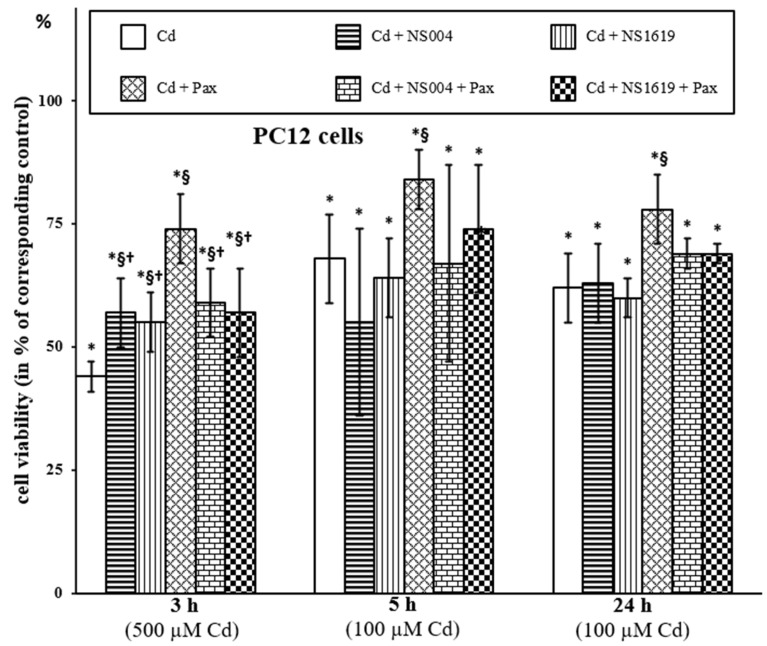
Action of BK(Ca) modulators on the survival of PC12 cells in the presence of Cd^2+^, depending on the duration of incubation and the metal concentration. Cell viability was expressed as a percentage of the respective untreated control (LDH release assay, see Section 4.2). The results are mean ± SEM (for n = 3–4 independent experiments). Statistical significance: * *p* < 0.05 with respect to the corresponding untreated control, ^§^ *p* < 0.05 with respect to Cd alone, ^†^ *p* < 0.05 with respect to Cd + Pax. The BK(Ca) modulators per se at used concentrations (10 µM—for NS004/NS1619 and 1 µM—for Pax) had no significant effects on the viability of the control PC12 cells at used durations of incubation.

Initially, we investigated the action of NS004 and NS1619 taken in a concentration of 10 µM on the survival of PC12 cells exposed to Cd^2+^ and estimated by the lactate dehydrogenase (LDH) release method (Figure 1). We obtained that the BK(Ca) openers themselves do not produce significant effects on the viability of the control cells under experimental conditions used, i.e., after 3, 5, and 24 h incubation with cells (data not presented). Interestingly, after 3 h of exposure, we found some mitigating action of NS004/NS1619 against Cd^2+^-induced cell viability loss, but this “protection” was negated after increasing the duration of incubation to 5 and 24 h (Figure 1). In contrast, the protective effect of Pax (1 µM) was significant at all times of incubation used; namely, Pax improved the cell viability, which was decreased by Cd^2+^, on average, by 30% in the presence of 500 µM Cd^2+^ after 3 h and by 16% in the presence of 100 µM Cd^2+^ after 5 and 24 h exposure (Figure 1). Importantly, in the simultaneous presence of Cd^2+^, Pax, and NS004/NS1619 in the medium during 5 or 24 h, the cell viability did not significantly differ from that observed in the presence of Cd^2+^ alone, whilst after 3 h of exposure, this difference was significant. In addition, the cell viability in the presence of Cd + Pax + NS004/NS1619 after 3 h of treatment was significantly less than that in the presence of Cd + Pax only. This means that the BK(Ca) openers counteracted the protective effect of Pax at all times of incubation used (3, 5, and 24 h). As also seen from Figure 1, the attenuating effect of NS004/NS1619 against Cd^2+^-induced cytotoxicity (observed only after 3 h exposure) was significantly lesser than that of Pax; moreover, this NS004/NS1619-induced “protection” was not affected by the presence of Pax in the medium.

#### 2.1.2. Effects of BK(Ca) Modulators on Respiration of PC12 Cells

Further, we studied the action of the BK(Ca) openers on the respiration of PC12 cells with the help of polarography (for details, see Section 4.2). The cellular respiration rates (or oxygen consumption rates, OCR) were monitored after exposure of PC12 cells to NS004/NS1619 for 3, 5, and 24 h and were expressed as a percentage of the maximal (fully uncoupled) respiration rate of the respective control cells, V_FCCP_(St3_u_) (Table 1). We found that NS004/NS1619 (10 µM) did not show any significant effects on the steady-state respiration rates at all times of incubation used, whilst after 3 h of exposure, these BK(Ca) activators significantly enhanced the resting respiration rates (in state 4_o_), i.e., after the addition of oligomycin (oligo)—a selective inhibitor of CV of mETC (F_1_F_0_-ATP synthase) (Table 1, see also Supplementary Material). In this regard, it is worthy to recall that cells utilize the oxygen uptake to maintain the steady-state level of ΔΨ_mito_ by compensating for (1) ATP synthesis by F_1_F_0_-ATPase and for (2) proton leak (i.e., so-called steady-state respiration). In turn, after oligo supplementation, which usually reduces cellular respiration rates, the portion of oxygen accumulation compensating only for the proton leak takes place (i.e., so-called resting respiration). We also revealed that after 5 and 24 h of exposure, NS004/NS1619 decreased significantly the maximal (fully uncoupled) respiration rate of PC12 cells (in state 3_u_), which was obtained after supplementation of an artificial protonophore FCCP (carbonyl cyanide *p*-trifluoromethoxyphenylhydrazone) to the respiratory assay medium (Table 1, see also Supplementary Material). It is known that after the addition of such chemical protonophores as FCCP or CCCP (carbonyl cyanide 3-chlorophenylhydrazone) to a suspension of cells or isolated mitochondria, the maximal rates of cellular/mitochondrial respiration (usually limited only by mETC capacity) are observed. As a result, the following time-dependent effects of NS004/NS1619 on respiration of PC12 cells were found: (1) the significant uncoupling effect observed after 3 h, consisting of the activation of the resting respiration rates at the unchanged maximal respiration rates (the early cellular effect of NS004/NS1619), and (2) the significant suppression of the maximal respiration rates after 5 and 24 h (Table 1). Importantly, as we have shown previously, Pax (i.e., BK(Ca) inhibitor), taken in its protective concentration (1 µM), significantly reduces the maximal respiration rates of PC12 cells after short-term (3–5 h) exposure, whereas these rates recover to control levels after prolonged (24 h) exposure to this compound, implying that reversible mETC impairment is the early cellular effect of Pax [35].

#### 2.1.3. Effects of BK(Ca) Modulators on ΔΨ_mito_ Changes in PC12 Cells

In the next experiments, an influence of different concentrations of BK(Ca) activators (NS004, NS1619) on ΔΨ_mito_ of PC12 cells in the absence and in the presence of different concentrations of Cd^2+^ was investigated with the help of JC-1 (5,5′,6,6′-tetrachloro-1,1′,3,3′- tetraethylbenzimidazolcarbocyanine iodide) (for experimental details, see Section 4.2) (Table 2).

Firstly, we studied the dose- and time-dependence of Cd^2+^ effects on ΔΨ_mito_ of the cells. We found that all [Cd^2+^] applied, except for 500 µM, did not produce significant action on ΔΨ_mito_ after 3 and 5 h exposure of cells to the heavy metal. In addition, 10 µM of Cd^2+^ did not induce significant changes in ΔΨ_mito_ of the PC12 cells at all used durations of incubation. In turn, 30 µM of Cd^2+^ significantly decreased ΔΨ_mito_ only after 24 h, while 50 and 100 µM of Cd^2+^ induced significant suppression of ΔΨ_mito_ starting from 16 h (Table 2). Secondly, the dose- and time-dependence of effects of NS004/NS1619 themselves on ΔΨ_mito_ in PC12 cells was monitored. We show that 10 µM of NS004/NS1619 did not produce significant effects on ΔΨ_mito_ at all durations of incubation used. At the same time, 30 µM of NS004/NS1619 was not effective at 3, 5, and 16 h exposure, whereas, after 24 h, this concentration of the BK(Ca) openers reduced ΔΨ_mito_, on average, by 11% (Table 2).

In addition, we studied the dose- and time-dependence of the combined action of the BK(Ca) activators and Cd^2+^ on ΔΨ_mito_ in PC12 cells (Table 2). We revealed that ΔΨ_mito_ changes in the simultaneous presence of 50 µM Cd^2+^ and 10 or 30 µM of NS004/NS1619 for 3 and 5 h were nonsignificant compared to the corresponding untreated controls. During 16 h of co-incubation of 50 µM Cd^2+^ with 10 or 30 µM of NS004/NS1619, there was a significant decrease of ΔΨ_mito_ in comparison with the respective untreated controls, but the changes in ΔΨ_mito_ were not significant compared to that observed in the presence of 50 µM Cd^2+^ alone (Table 2). After 24 h, the ΔΨ_mito_ values in the simultaneous presence of 50 µM Cd^2+^ and 10 µM NS004/NS1619 significantly decreased compared to the corresponding untreated controls, but they were nonsignificant compared to that obtained in the presence of 50 µM Cd^2+^ only. Notably, the significant enhancement of ΔΨ_mito_ decline during co-incubation of 50 µM Cd^2+^ with 30 µM NS004/NS1619 for 24 h was found (Table 2). In the case of 100 µM Cd^2+^, the significant ΔΨ_mito_ decrease (compared to control) was observed already after 5 h, however, only during co-administration of this heavy metal with 30 µM NS004/NS1619. After 24 h, the ΔΨ_mito_ loss in the presence of 100 µM Cd^2+^ was prominent, and the co-administration with BK(Ca) openers did not significantly influence on it (Table 2). As to 500 µM Cd^2+^, the significant ΔΨ_mito_ decrease (compared to the respective control) was found already after 3 h; in addition, in this case, there was no significant effect of NS004/NS1619 at used concentrations on ΔΨ_mito_ of Cd^2+^-treated cells (Table 2).

#### 2.1.4. Effects of BK(Ca) Modulators on ROS Production in PC12 Cells

As we have found previously, Cd^2+^ induces dose- and time-dependent changes in ROS formation of PC12 cells; furthermore, these changes are crucial for the cytotoxic effect of the heavy metal [14,41]. Some time ago, we also revealed that Pax (taken in a concentration of 1 µM) not only protected partially against Cd^2+^-induced viability loss in PC12 cells but also significantly reduced the intracellular ROS production rise induced by Cd^2+^ [35]. In the present work, we studied effects of BK(Ca) openers (NS004, NS1619) on ROS generation of PC12 cells in the absence and in the presence of Cd^2+^ or/and Pax using the redox-sensitive probe, 2′,7′-dichlorodihydrofluorescein diacetate (DCFH_2_-DA) (for experimental details, see Section 4.2). We show that after 30 min, the intracellular ROS formation in the presence of NS004 or NS1619 (10 µM) did not differ significantly from the corresponding control, while it significantly increased after 3 h, on average, by 40–50% compared with the control; furthermore, in this case, the ROS production rise was significantly reduced by Pax, on average, by 30–40% (Table 3). In addition, after 30 min of exposure, NS004 or NS1619 did not significantly affect the Cd^2+^-induced ROS production increase, in contrast to the significant reducing effect of Pax observed during this duration of incubation. Interestingly, the maximal decrease in ROS production of Cd^2+^-treated PC12 cells after 30 min was found during the simultaneous co-incubation of these cells with Pax and the BK(Ca) openers (Table 3). After 3 h, the ROS formation of (Cd^2+^ *plus* NS1619)-treated PC12 cells significantly increased in comparison with that observed in the presence of NS1619 alone or Cd^2+^ alone; moreover, this enhancement of the intracellular ROS production was sensitive to Pax, namely, it significantly decreased in the presence of the BK(Ca) blocker (Table 3). Of note, the intracellular ROS production during co-incubation of Cd^2+^, NS1619, and Pax was 132 ± 9% (as a percentage of the control) that was significantly less than in the presence of Cd^2+^ alone (157 ± 10%) or Cd^2+^ *plus* NS1619 (179 ± 1%), but did not significantly differ from that in the presence of Cd^2+^ *plus* Pax (135 ± 8%). The close results were obtained after 3 h for NS004.

### 2.2. Action of BK(Ca) Modulators on AS-30D Cells in the Absence and in the Presence of Cd^2+^

We also investigated the effects of NS004 and NS1619 on viability, respiration, ΔΨ_mito_, and ROS generation in the absence and in the presence of Pax and/or Cd^2+^ using another type of rat cell line, namely ascites hepatoma AS-30D cells.

#### 2.2.1. Effects of BK(Ca) Modulators on Viability of AS-30D Cells

**Figure 2:**
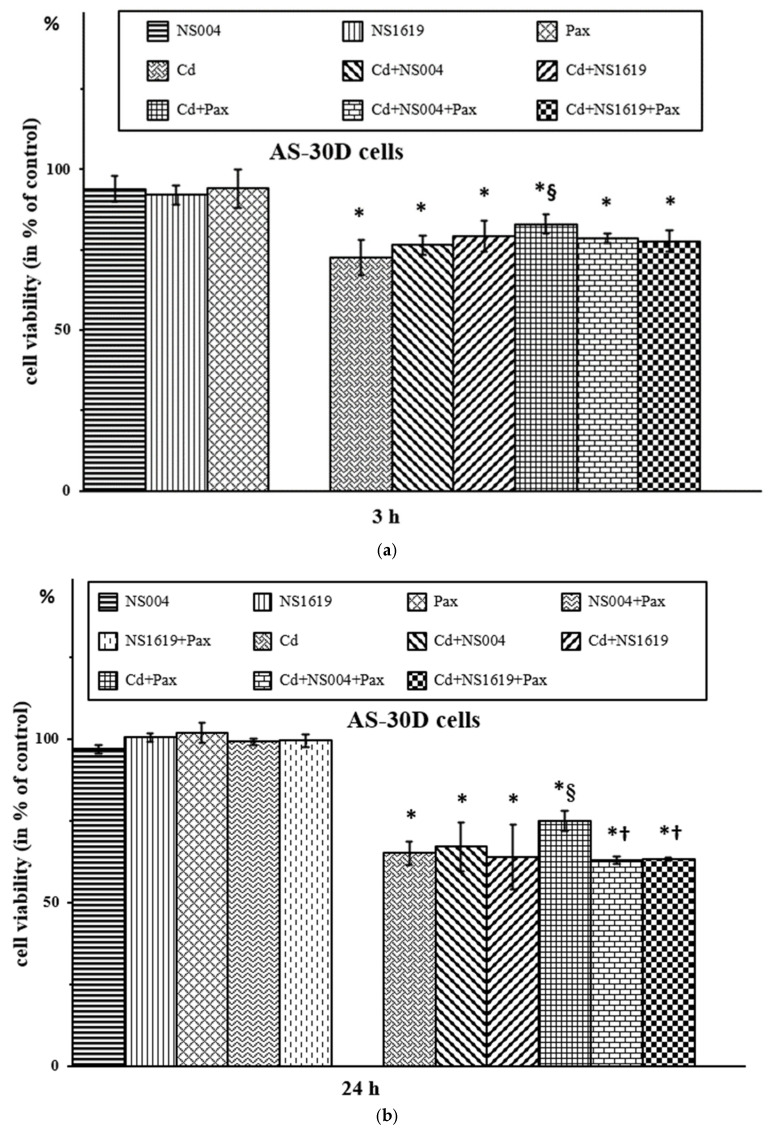

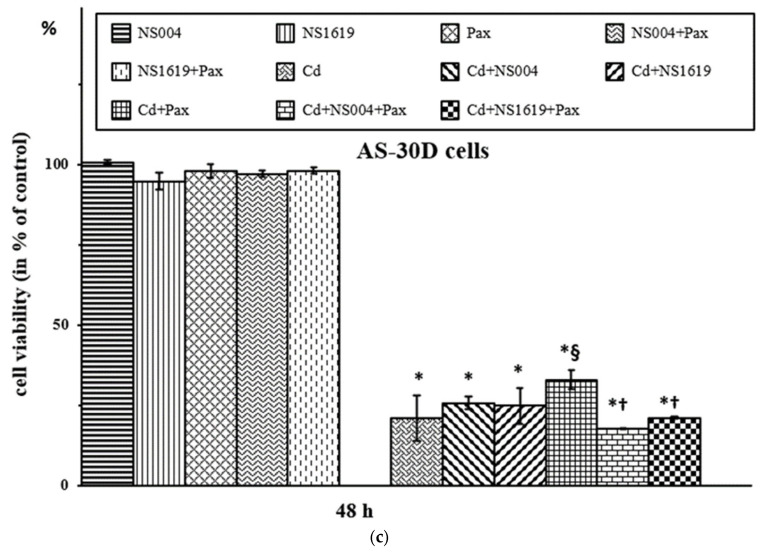
Effects of BK(Ca) modulators on viability of AS-30D cells measured by TB exclusion test (showing a loss of plasma membrane integrity) in the absence and in the presence of different concentrations of Cd^2+^ at different times of exposure. (**a**): 3 h, (**b**): 24 h, and (**c**): 48 h; [Cd^2+^] = 100 µM (3 h) or 50 µM (24 and 48 h). The concentration of NS004/NS1619 was 10 µM. The results shown are mean ± SEM (for n = 3–9 independent experiments). Statistical significance: * *p* < 0.05 with respect to the corresponding untreated control, ^§^ *p* < 0.05 with respect to Cd alone, ^†^ *p* < 0.05 with respect to Cd + Pax.

In our previous works [35,49], we showed that the BK(Ca) blocker, Pax, was protective in part against Cd^2+^-induced necrosis of AS-30D cells, but it did not produce a significant effect on the cell apoptosis caused by this heavy metal ion. In addition, we found that BK(Ca) activators, NS004 and NS1619, themselves, produced apoptosis of AS-30D cells [49]. In these works, the trypan blue (TB) exclusion method showing a loss of plasma membrane integrity was used to monitor (in some approximation) cell necrosis, while DNA fragmentation estimated by the propidium iodide (PI) staining method with the following quantification of the resulting sub-G_1_ fraction was used as a widely accepted test for cell apoptosis (see also Section 4.3). In the current study, using the same approach, we found that after 3 (a), 24 (b), or 48 h (c) (Figure 2), NS004 and NS1619 taken in 10 µM concentration did not produce the significant effect on viability of AS-30D cells measured by the TB exclusion test and estimated as a percentage of the corresponding control that reflected the percent of viable cells, i.e., TB-negative. We found also that there was no significant effect of the BK(Ca) activators on the Cd^2+^-induced necrosis of AS-30D cells (Figure 2). However, in both cell types, NS004/NS1619 counteracted the mitigating effect of Pax against Cd^2+^-induced necrosis, namely after 3, 5, and 24 h in PC12 cells (Figure 1) and after 24 and 48 h in AS-30D cells (Figure 2b,c). After 3 h, NS004/NS1619 did not show a significant effect on the protection produced by Pax against the Cd^2+^-induced viability decline of AS-30D cells (Figure 2a). Interestingly, in PC12 cells, the significant effects of NS004/NS1619 on the cell viability in the presence of Cd^2+^ were observed already from 3 h (both the protective and the antagonistic ones) (Figure 1).

**Figure 3:**
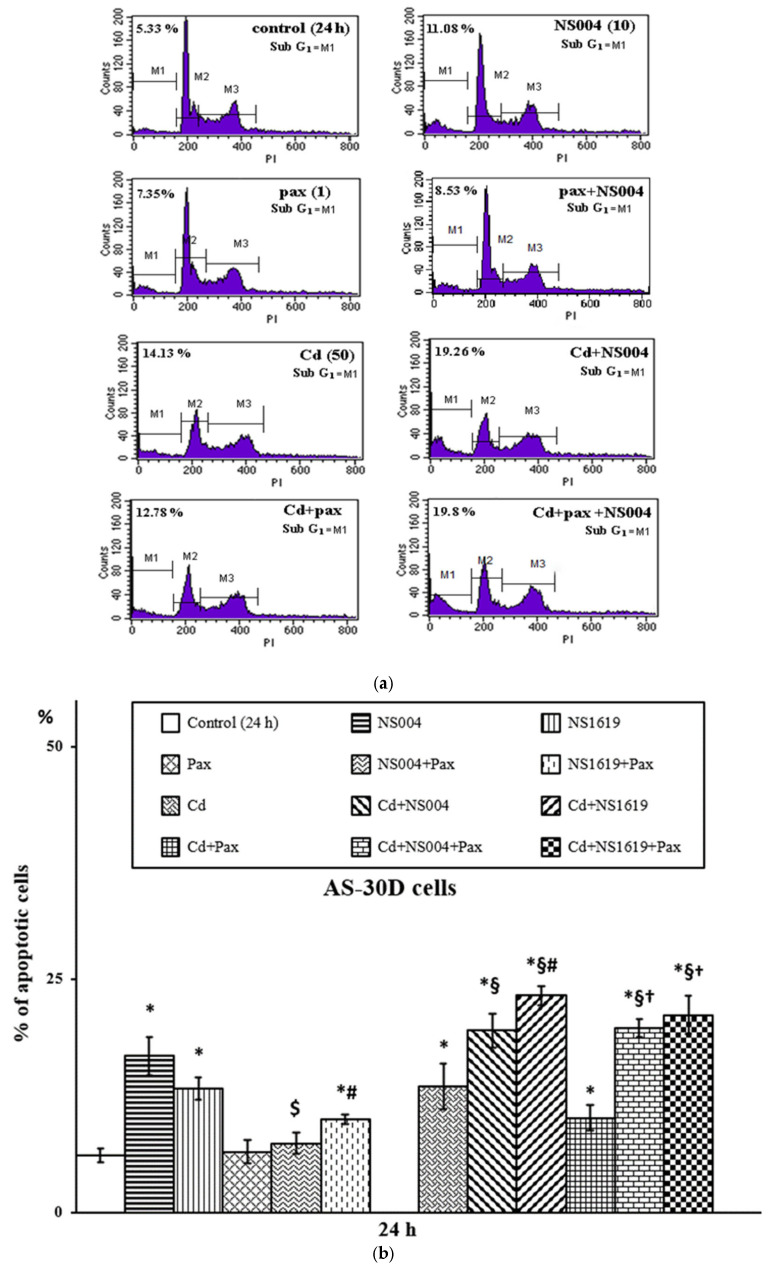
Apoptosis of AS-30D cells in the presence of BK(Ca) modulators and/or Cd^2+^. The cells were incubated without (control) or with NS004/NS1619 and/or Cd^2+^ (in the absence or presence of Pax), stained with propidium iodide, and analyzed by flow cytometry (see Section 4.3). When used, the concentration of NS004/NS1619 was 10 µM, Pax—1 µM, and Cd^2+^—50 µM (24 h). (**a**) The percent of the sub-G_1_ fraction, characteristic for apoptotic cells, is indicated in the upper left corner of each panel. The results shown are representative of no less than three independent experiments; (**b**) The data presented are mean ± SEM (n = 3–4). Statistical significance: * *p* < 0.05 with respect to the corresponding untreated control, ^$^ *p* < 0.05 with respect to NS004, ^#^ *p* < 0.05 with respect to NS1619, ^§^ *p* < 0.05 with respect to Cd alone, ^†^ *p* < 0.05 with respect to Cd + Pax.

Additionally, we show that the BK(Ca) openers used at the same concentrations (i.e., 10 and 30 µM) induced apoptosis of AS-30D cells both after 24 (Figure 3) and 48 h. Importantly, the NS004/NS1619-induced apoptosis of AS-30D cells was found to be sensitive to Pax; namely, it was significantly reduced in the presence of this BK(Ca) inhibitor (Figure 3). We also found that in the simultaneous presence of Cd^2+^ and NS004 or NS1619 in the medium, the number of apoptotic cells significantly increased compared with Cd^2+^ alone; the percent of apoptotic cells observed in the presence of Cd^2+^ *plus* NS1619 was significantly higher than that in the presence of NS1619 alone (Figure 3b). As also seen, there is a tendency to decrease the Cd^2+^-induced apoptosis in the presence of Pax, in contrast to the enhancing effect of the BK(Ca) openers on it (Figure 3b). Of note, in the current study, we revealed that Pax had no significant action on (Cd^2+^ *plus* NS004/NS1619)-induced apoptosis of AS-30D cells, even though it exhibited the significant reducing effect on the NS004/NS1619-induced apoptosis of the cells (Figure 3). Overall, this means that there is an interplay between the effects of BK(Ca) modulators and Cd^2+^ when used together, i.e., these compounds are capable of influencing each other’s effects.

#### 2.2.2. Effects of BK(Ca) Modulators on Respiration of AS-30D Cells

We monitored the effects of the BK(Ca) activators on respiratory rates of AS-30D cells; namely, we measured OCR in the steady state (basal respiration rate), in the resting state (St4_o_ respiration rate), and after addition of CCCP (St3_u_ respiration rate) during incubation of these cells with NS004 or NS1619 (10 µM) for 3, 24, and 48 h. We show that under the used conditions, these BK(Ca) openers had no significant effect on all respiratory rates under study. In particular, after 3 and 48 h of treatment of cells with the BK(Ca) activators, the respiratory rates (counted as a percentage of V_CCCP_(St3_u_) of the corresponding control) were the following (n = 3): (1) basal rates—50 ± 2 (control_3h_), 45 ± 5 (NS004), 46 ± 2 (NS1619), (2) St4_o_ rates—23 ± 4 (control_3h_), 19 ± 1 (NS004), 20 ± 3 (NS1619), (3) St3_u_ rates—100 (control_3h_), 90 ± 10 (NS004), 96 ± 6 (NS1619)—3 h; (1) basal rates—65 ± 4 (control_48h_), 56 ± 5 (NS004), 61 ± 4 (NS1619), (2) St4_o_ rates—19 ± 3 (control_48h_), 16 ± 3 (NS004), 18 ± 3 (NS1619), (3) St3_u_ rates—100 (control_48h_), 97 ± 8 (NS004), 100 ± 4 (NS1619)—48 h.

As follows from the results obtained, in AS-30D cells, BK(Ca) openers, NS004 and NS1619, do not show any uncoupling action at all durations of incubation used, in contrast to that observed in their presence in PC12 cells after 3 h (Table 1) or after the same time of incubation with diazoxide (mK(ATP) opener) in AS-30D cells [33,34]. They also do not have the significant suppressing effect on the maximal (St3_u_) respiration rates of AS-30D cells, in contrast to that found in the case of PC12 cells after prolonged exposure with these openers (Table 1). As to Pax (BK(Ca) blocker), its effect was the same in both types of cells; namely, it did not affect the basal and resting respiration rates at all times of exposure but exhibited the significant reversible decrease of the maximal respiration rates after short-term treatments [35].

#### 2.2.3. Effects of BK(Ca) Modulators on ΔΨ_mito_ Changes in AS-30D Cells

**Figure 4:**
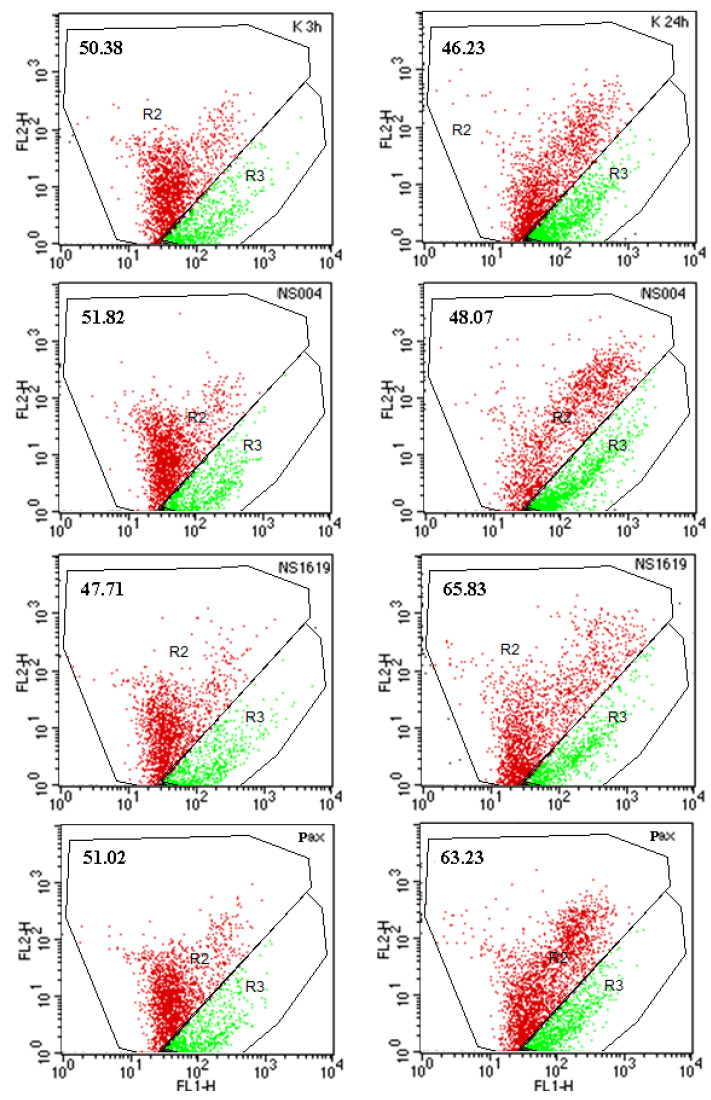
Effect of BK(Ca) modulators on mitochondrial membrane potential (ΔΨ_mito_) of AS-30D cells under different durations of incubation with the drugs. The cells were incubated without (control) or with NS004/NS1619 (10 μM) or Pax (1 μM) for 3 (**left panel**) or 24 h (**right panel**) before staining with JC-1. This cell-penetrating dye accumulates within mitochondria, maintaining high ΔΨ_mito_, and changes its emission fluorescence from green to red, which can be monitored by flow cytometry. The percent of cells with red (R2) and green (R3) JC-1 fluorescence, reflecting high and low ΔΨ_mito_, correspondingly, was determined. A typical experiment out of at least three independent ones is shown (R2 is indicated in the upper left corner of each panel). R2 (mean ± SEM at 0.95 probability level, n = 3–4) were the following (in %): 3 h—57 ± 6 (control_3h_), 49 ± 14 (NS004), 44 ± 13 (NS1619), 46 ± 6 (Pax); 24 h—53 ± 7 (control_24h_), 49 ± 2 (NS004), 60 ± 6 (NS1619), 56 ± 7 (Pax).

In the next experiments, we studied the action of the BK(Ca) modulators under test on ΔΨ_mito_ of AS-30D cells depending on the duration of incubation of cells with the drugs (Figure 4). We show that BK(Ca) openers, NS004 and NS1619, taken in a concentration of 10 µM, did not produce a significant effect on ΔΨ_mito_ of AS-30D cells after both 3 (Figure 4, left panel) and 24 h (Figure 4, right panel). As to Pax, its “protective” concentration, namely 1 µM, had no significant action on ΔΨ_mito_ of AS-30D cells after 3 and 24 h as well (Figure 4) and [35]. Moreover, this “mitigating” concentration of Pax did not exhibit significant action on the changes of ΔΨ_mito_ observed in the presence of Cd^2+^ in both types of cells under study [35].

#### 2.2.4. Effects of BK(Ca) Modulators on ROS Production in AS-30D Cells

**Figure 5:**
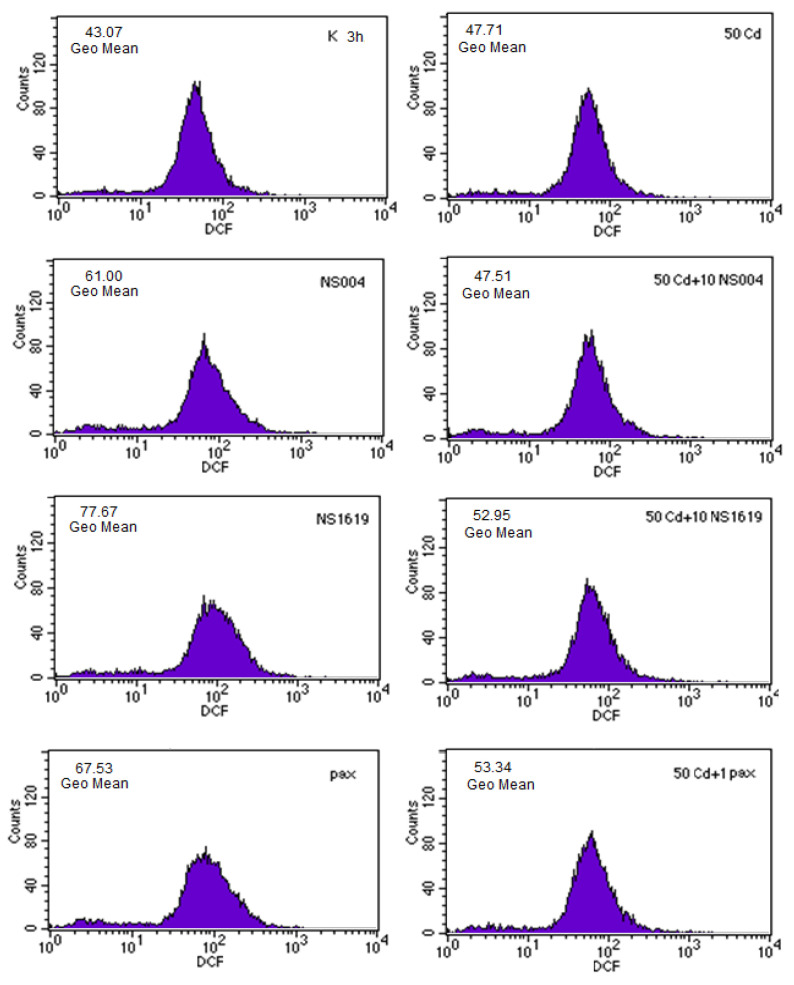
Action of BK(Ca) modulators on ROS production by AS-30D cells in the absence and in the presence of Cd^2+^ measured by flow cytometry with the aid of DCFH_2_-DA as the redox-sensitive probe. AS-30D cells were incubated without (control) or with NS004/NS1619 or Pax for 3 h in the absence (left panel) or presence of Cd^2+^ (right panel) before staining with DCFH_2_-DA (for details, see Section 4.3). When added, concentrations (in µM) of NS004/NS1619 were 10, Pax—1, and Cd—50. The overall ROS generation is depicted as the geometric mean of the total green fluorescence of the oxidation product of DCFH_2_ and shown in arbitrary units (a.u.) in the upper left corner of each panel. The figure is representative of 3–4 independent experiments.

Further, we have conducted a set of experiments testing the action of the BK(Ca) modulators on ROS formation of AS-30D cells in the absence and in the presence of Cd^2+^, the results of which are presented in Table 4 and Figure 5; in particular, we studied the effects of NS004 and NS1619 on intracellular ROS production in the absence and in the presence of two concentrations of Cd^2+^: 50 (Figure 5) and 100 μM (Table 4) after 50 min, 3 h, and 24 h of incubation with the drugs. It has been found that the effects of these BK(Ca) activators depend heavily on the duration of incubation with cells. As seen from Table 4, NS004/NS1619 (10 μM) per se decreased ROS generation of the control AS-30D cells after 50 min, whereas it increased it after 3 and 24 h of treatment. Notably, after 50 min exposure of AS-30D cells to NS004/NS1619 and Cd^2+^, the intracellular ROS formation became close to the control level and was significantly less than that observed in the presence of Cd^2+^ alone (Table 4). After 3 h, the intracellular ROS production in the presence of Cd^2+^, or NS004/NS1619, or both together was significantly higher than in the control; in addition, there were no significant differences between its values when Cd^2+^ or the BK(Ca) openers were used separately or together (Table 4).

It is important to emphasize that in the present study, we found that in both types of cells, NS004/NS1619 exerted the temporary “protection” against Cd^2+^-induced ROS rise at early times of incubation, i.e., after 50 min in AS-30D cells (Table 4) and after 30 min in PC12 cells, where they enhanced the ROS decrease during coincubation with Cd^2+^ and Pax (Table 3). Moreover, this was the early cellular effect of the BK(Ca) activators. In the case of AS-30D cells, this could be partially explained by the mitigating action of NS004/NS1619 on the intracellular ROS production in the control, namely by their reducing effect on the ROS formation of the control cancer cells at short durations of incubation (Table 4). In the case of PC12 cells, this is highly likely connected in part with the stimulation of the resting respiration rate of the cells in the presence of the BK(Ca) openers at short-time exposures, i.e., up to 3 h (Table 1). It should be noted also that Pax per se (taken in the “protective” concentration) significantly increased the intracellular ROS formation of AS-30D cells starting from 3 h and did not decrease its rise in the presence of Cd^2+^ at all times of incubation (Table 4 and Figure 5), contrary to that observed in PC12 cells, where Pax itself did not affect the ROS production in the control and significantly decreased the ROS rise in the presence of Cd^2+^, or NS004/NS1619, or both together (Table 3) (see also [35]).

## 3. Discussion

**Figure 6:**
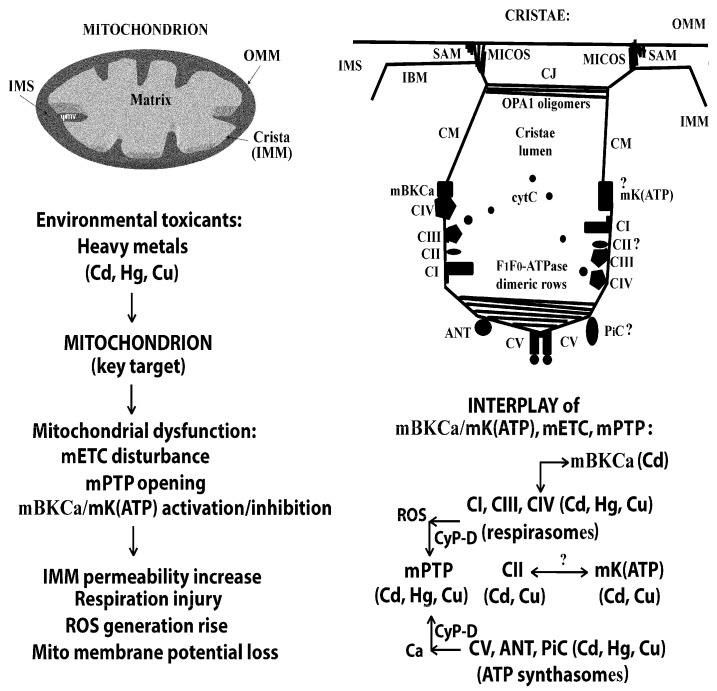
Molecular mechanisms of cytotoxicity of heavy metals: mitochondrial dysfunction and oxidative stress, and the interplay between mETC, mPTP, and mBK(Ca)/mK(ATP). The model presented is modified from Ref. [34].

At present, it has been shown that heavy metal ions, and Cd^2+^ in particular, suppress the viability of different types of cells, including PC12 and AS-30D cells used in the current work, in which Cd^2+^ evokes both necrosis and apoptosis and induces mitochondrial oxidative stress and mitochondrial dysfunction mediated by mETC disturbance and mPTP induction (Refs. [12,13,14,19,20,21,22,23,38,61,62,63,64,65] and references therein; see also Figure 6). As found, selective cellular/organellar K^+^ channels contribute to many essential cellular functions, including cell excitability and contractility, Ca^2+^ homeostasis, cellular signaling, and regulation of cell death and adaptive responses of different types; in addition, mitochondrial selective K^+^ channels, such as mBK(Ca) and mK(ATP), play the crucial role in the regulation of mitochondrial function, energy metabolism, and cell destiny in general [28,34,47,50,66]. Previously, we found that BK(Ca) modulators affected Cd^2+^-induced cytotoxicity; namely, Pax (i.e., BK(Ca) inhibitor) mitigated the toxic effects of this heavy metal, causing the reduction of the number of necrotic cells and the intracellular ROS production in the presence of Cd^2+^, whereas NS004 and NS1619 (i.e., BK(Ca) openers) themselves induced apoptosis both in the absence and in the presence of Cd^2+^, and the number of apoptotic cells increased during their co-administration with this toxic metal [35,49]. In the present work, we tested our hypothesis about the possible participation of BK(Ca)s in the above events using a pharmacological approach, namely, by studying the relationship between the effects of NS004/NS1619, Pax, and Cd^2+^.

### 3.1. Synopsis of the Results on the Effect of BK(Ca) Modulators on PC12 and AS-30D Cells Exposed to Cd^2+^

In the present study, we investigated the action of different concentrations of NS004/NS1619 on the functioning of cells from two rat cancer lines, PC12 and AS-30D, and estimated the effects of these BK(Ca) activators on cell survival, cellular respiration, ΔΨ_mito_, and intracellular ROS production in the absence and in the presence of Pax and/or Cd^2+^. In consequence, the following important findings were obtained.

Firstly, we found that in PC12 cells, NS004/NS1619 at used concentrations produced (i) the significant effects on cellular respiration depending on the duration of incubation, activating the resting respiration rate at 3 h and partially suppressing the maximal (fully uncoupled) respiration rate after 5 and 24 h of incubation (Table 1), and (ii) the significant decrease of ΔΨ_mito_, depending on concentration and time used, in the absence and the presence of different concentrations of Cd^2+^ (Table 2). In AS-30D cells, these BK(Ca) activators induced the time- and dose-dependent apoptosis, which significantly decreased in the presence of Pax (Figure 3). In both types of cells, NS004/NS1619 counteracted the attenuating effect of Pax against Cd^2+^-induced necrosis (Figure 1 and Figure 2) and significantly increased the intracellular ROS formation at 3 h (Figure 5, Table 3 and Table 4), which, in the case of PC12 cells, was significantly reduced by Pax (Table 3). In addition, in PC12 cells, the significant enhancement of the intracellular ROS production in the simultaneous presence of Cd^2+^ and NS004/NS1619 in the medium was observed after 3 h compared to Cd^2+^ or NS004/NS1619 alone; moreover, Pax decreased significantly the ROS rise both in the presence of Cd^2+^ and Cd^2+^ together with NS004/NS1619 (Table 3). In total, this suggests that BK(Ca)s may somehow be involved in the observed cellular events.

Secondly, in both types of cells, NS004/NS1619 (10 µM) exerted the temporary “protection” against Cd^2+^-induced ROS rise, reducing the intracellular ROS production at early times of incubation, i.e., after 50 min in AS-30D cells (Table 4) and after 30 min in PC12 cells (Table 3); in particular, in the latter case, NS004/NS1619 enhanced the reduction of ROS levels upon simultaneous incubation with Pax and Cd^2+^ (Table 3). Of note, after 3 h, NS004 or NS1619 taken at the same concentration themselves induced the significant ROS rise in both types of cells (Figure 5 and Table 3 and Table 4); in addition, this concentration of the BK(Ca) activators did not affect ΔΨ_mito_ after 3 and 24 h in AS-30D cells (Figure 4) and after 3, 5, 16, and 24 h in PC12 cells (Table 2). This indicates that the significant changes in the intracellular ROS production caused by NS004/NS1619 in the absence or in the presence of Pax and/or Cd^2+^ are the early cellular effects of the BK(Ca) openers.

Thirdly, we show that two kinds of protection by BK(Ca) modulators against Cd^2+^-induced cell viability decline occurred: (1) the sustained mitigating effect of Pax (i.e., BK(Ca) blocker) observed in both types of cells at all used durations of incubation, namely after 3, 5, 24, and 48 h of exposure (Figure 1 and Figure 2, and [35,49]) and (2) the transient attenuating effect of NS004/NS1619 (i.e., BK(Ca) activators) observed in PC12 cells at 3 h of incubation (Figure 1). Noteworthily, the activation of the resting respiration rates by NS004/NS1619 at short-time incubations in PC12 cells (3 h, Table 1) may partially explain the temporary “protection” by NS004/NS1619 against the cell viability decrease in the presence of Cd^2+^ (3 h, Figure 1), as well as the enhancement of the decline of intracellular ROS production in Cd^2+^-treated PC12 cells co-incubated with NS004/NS1619 and Pax (30 min, Table 3). In turn, from 5 to 24 h of incubation, NS004 and NS1619 themselves did not stimulate the resting respiration rates but significantly decreased the maximal respiration rates of the control PC12 cells (Table 1); in addition, these BK(Ca) openers enhanced ROS production rise (Table 3) and ΔΨ_mito_ decline (Table 2) of Cd^2+^-treated PC12 cells. Notably, this may partially explain the elimination of the following two protective effects taking place in the presence of BK(Ca) modulators: (1) the protective effect of NS004/NS1619 against Cd^2+^-induced decrease in cell survival and (2) the protective effect of Pax on cell viability in the simultaneous presence of Cd^2+^ and NS004/NS1619, both of which are observed after 5 and 24 h in PC12 cells (Figure 1). It is worthy to remind also that in both types of cells, Pax per se (used in the protective concentration) does not affect the resting respiration rates and ΔΨ_mito_ values at all durations of incubation applied but significantly increases the ROS production in AS-30D cells after 3 and 24 h (Table 4); Pax significantly decreases the maximal respiration rates in both types of cells after short-term (3–5 h) durations of incubation, which are restored up to the control levels after long-term (24–48 h) incubations [35,49]. This means that the sustained reversible decrease of mETC activity by Pax is the early cellular effect of the compound. Taken together, this implies that mETC and changes in intracellular ROS levels are among the key players in the molecular mechanism(s) of action of the BK(Ca) modulators.

Fourthly, we revealed the interplay between the effects of Cd^2+^, NS004/NS1619, and Pax; in particular, we found that the changes in the intracellular ROS production/ΔΨ_mito_/cell viability induced by the corresponding concentrations of Cd^2+^ and the BK(Ca) modulators were affected upon their combined application in PC12 (Figure 1 and Table 2 and Table 3) and AS-30D cells (Figure 2 and Figure 3 and Table 4). In other words, the significant mutual influence of Cd^2+^ and BK(Ca) modulators on the effects of each other takes place. Importantly, this suggests that Cd^2+^, a known hazardous environmental pollutant, may interfere with the function of BK(Ca)s in the cell.

Thus, all of this together points to the involvement of ROS, mETC, and, very likely, BK(Ca)s (most probably, mBK(Ca)) in the mechanisms of modulation of Cd^2+^-induced cytotoxicity by the BK(Ca) modulators applied. However, it is important to add that the above conclusions regarding the role of BK(Ca)s are based on pharmacological tools (NS004/NS1619, Pax), and these compounds are known to have off-target effects (see Section 1, p. 3). Therefore, to strengthen the arguments and further elucidate the molecular mechanisms, it is necessary to obtain direct evidence of the involvement (or non-involvement) of BK(Ca)s in the observed phenomena, as well as to clarify the relationship between mETC and the channels under the conditions used, which is what we intend to do in our future studies (see Section 5).

### 3.2. On the Relationship Between mBK(Ca), mETC, and mPTP in the Absence and in the Presence of Cd^2+^

The results obtained in the present study are in good agreement with data available in the literature in the field. As already mentioned in the Introduction, mBK(Ca) activation is generally protective of cellular function; nevertheless, BK(Ca) openers are able to induce or enhance cell death under different conditions, especially in cancer cells (refs. [28,35,36,37,47,49,50,51,52,53,54,55,56,66] and references therein). It should be recalled that mBK(Ca) activation modulates matrix volume, respiration rates, ROS formation, ΔΨ_mito_ changes, and Ca^2+^ overload; in addition, this channel is shown to be involved in regulation of MPT pore opening [28,47,67,68,69,70,71,72,73,74,75,76].

It is worth noting that there is no consensus in the field in relation to the effects of mBK(Ca) on ROS production. Although there are numerous reports demonstrating that mBK(Ca) activation results in decreased ROS levels and that the use of inhibitors of this channel causes an increase in intracellular ROS formation [28,47,66,75,77], there is evidence to suggest the opposite. Specifically, addition of NS11021 (BK(Ca) opener) to isolated and I/R injury-induced ventricular myocytes resulted in increased ROS production and cell survival, which was abolished by Pax or tempol [78]. Elevated ROS levels following mBK(Ca) activation were also detected in another liver cancer cell line [79]. It is believed that the ambivalence of ROS responses observed after mBK(Ca) activation may be related to possible coupling between the channel and sites of ROS formation. Thus, if the channel is coupled with CI of mETC (reverse electron transport, RET), ROS production decreases after its activation. In turn, when mBK(Ca) is coupled to CIII of mETC (forward electron transport, FET), ROS growth occurs after channel activation (refs. [47,80] and references therein). It is also known that the relative contribution of these two mETC complexes to ROS generation can vary significantly between cells. In this regard, it should be noted that we have previously established that Cd^2+^-induced cytotoxicity is associated with ROS produced by mETC, mainly CIII of mETC, since the formation of ROS induced by this heavy metal was significantly reduced in the presence of stigmatellin in both types of cells used; moreover, this selective CIII (III_Qo_) inhibitor significantly increased the viability of Cd^2+^-treated PC12 and AS-30D cells [14,38,41]. At the same time, rotenone, a selective inhibitor of CI (I_Q_) of mETC, had no significant effect on the viability of Cd^2+^-treated PC12 and AS-30D cells; furthermore, it significantly decreased the viability of the control AS-30D cells [14,38].

It is important to note that BK(Ca) activators can both suppress [81,82,83,84] and enhance [85,86,87] cellular and mitochondrial respiration. In particular, such BK(Ca) openers as NS004 and NS1619 are able to inhibit mETC in various types of cells [81,82,83,84]. At the same time, in hippocampal slice cultures exposed to glutamate, preincubation with NS1619 showed an increase in basal respiration [86]. In addition, there is evidence that in guinea pig heart mitochondria, such a highly selective NS compound as NS11021, taken in the nM range, reduced the St4 respiration rate with an unchanged St3 respiration rate, whereas 1 μM of NS11021 significantly increased the resting respiration rate without affecting ADP-stimulated respiration [88]. In turn, activation of BK(Ca) by NS11021 or rottlerin during ischemic cardioplegic arrest increased maximal (FCCP-uncoupled) respiration rate, i.e., V_FCCP_(St3_u_), and rottlerin-modulated effects on respiration were significantly reduced by Pax; moreover, the use of these BK(Ca) openers promoted the formation of respiratory mitochondrial supercomplexes (mSCs), consisting predominantly of CI, CIII, and CIV of mETC [85]. NS11021 protected normal rat kidney proximal tubule cells from cold storage-induced mitochondrial injury in vitro by attenuating superoxide production, depolarization, and mitochondrial respiratory dysfunction. Notably, 1 μM of this specific BK(Ca) activator partially protected against the decrease in ADP-stimulated (St3) respiration rates at CI, CII, and CIV levels and caused complete protection at the CIII level [87]. Recently, it has been shown also that NS1619 inhibits dose-dependently the respiration and oxidative phosphorylation of mouse skeletal muscle mitochondria energized by Glu/Mal or Succ, i.e., substrates of CI and CII, respectively, and its effect is based on the inhibition of CI, CIII, and CIV activities of mETC as well as F_1_F_0_-ATPase and is accompanied by a dose-dependent decrease in ΔΨ_mito_; additionally, NS1619 significantly decreased the ability of these mitochondria to uptake and retain Ca^2+^ in the matrix but evoked the decrease in H_2_O_2_ production in the presence of Glu/Mal [89]. In sum, all of this means that the effect of BK(Ca) activators on cellular respiratory function is highly dependent on the cell type, concentration, and conditions used.

Intriguingly, some researchers believe that the primary mitochondrial effect of NS1619 is the inhibition of CI of mETC rather than the activation of mBK(Ca), and that its neuroprotective effect observed during preconditioning is a consequence of ROS generation through the suppression of mETC complexes and stimulation of the corresponding signaling pathways [83]. There are also some other cellular targets of NS1619, among them CV of mETC (F_1_F_0_-ATPase) [84,89]. In particular, it has been shown that ATP synthase in endothelial cells is inhibited by this BK(Ca) opener in an oligomycin-like manner [84]. The inhibition of SERCA and CI of mETC are involved in pleiotropic effects of NS1619 on the endothelial cells as well [84]. Recent studies indicate that NS1619 effects in muscle tissues are complex, involving not only direct channel activation but also potentially modulating mitochondrial respiratory complexes, affecting mitochondrial gene expression, and impacting mitochondrial function in a disease-dependent manner. Specifically, in mouse Duchenne muscular dystrophy (DMD) models, NS1619 can normalize mitochondrial function and structure, but it has detrimental effects on healthy muscle mitochondria by inhibiting their function and disrupting structure [89,90,91].

In addition, there is data that cytochrome c oxidase (CIV of mETC) is able to connect with regulatory subunits of mBK(Ca) [92,93]. Specifically, it has been found that cyt c oxidase subunit 1 directly binds to the regulatory β1 subunit of this channel in cardiac cells [92]; physical association of the regulatory β4 subunit of mBK(Ca) with cyt c oxidase has been demonstrated in the human glioblastoma cell line U-87 MG [93]. Moreover, it has been revealed that activation of mETC by energetic substrates (NADH, Succ, Glu/Mal, or ascorbate/TMPD) reduces the probability of mBK(Ca) opening, and this is inhibited by specific mETC blockers (rotenone, antimycin A, and cyanide) [93]. These findings imply that mBK(Ca) is regulated by CIV of mETC and that a redox signal is likely transferred from the respiratory chain to the channel via cyt c oxidase. Taken together, all of this indicates the existence of a structural-functional relationship between mETC and mBK(Ca) (Figure 6), the molecular mechanisms of which, however, have not yet been elucidated [47,66,92,93].

Notably, the potential “protein” partnership of BK(Ca)s of different types was studied as well. Interestingly, using a proteomic approach on rat cardiomyocytes, it was found that the largest number of mitochondrial proteins interacting with cardiac mBK(Ca) belong to the oxidative phosphorylation system (OXPHOS), among which are subunits of four mETC complexes, subunits of ATP synthase, as well as subunits of adenine nucleotide translocase (ANT) and phosphate transporter (PiC) [80,94]. Furthermore, a novel interaction between mBK(Ca) and ANT was discovered in HEK293T cells, namely their association via the transmembrane domain of the channel. As a result, Toro and coauthors (2017) suggested that in the IMM, mBK(Ca) may directly interact with ANT, which can be in conjunction with ATP synthasome or separate, or via a not yet identified intermediary partner [94]. It is also important to add that Du et al. (2020), using mitochondria of HEK and PC12 cells transfected with mutant BK(Ca)s carrying a mutation affecting the selectivity filter of the channel, showed that this mutation resulted in a selective loss of BK(Ca)s in the mitochondrial membrane; moreover, these researchers revealed a loss of mitochondrial content ranging from the loss of voltage-dependent anion channel (VDAC) proteins to a decrease in each component of OXPHOS, which ultimately caused mitochondrial dysfunction in the cells [76]. All of this means that mBK(Ca) plays a critical role in maintaining mitochondrial structure, function, and content [47,76].

It should be noted that, in recent years, significant progress has been made in understanding of the ultrastructural organization of mitochondria. As is known, IMM is folded in parallel lamellar cristae, and its non-invaginated part, i.e., the inner boundary membrane (IBM), forms a cylindrical sandwich with the outer mitochondrial membrane (OMM). Crista membranes (CMs) are connected to IBM at crista junctions (CJs) of mitochondrial cristae organizing system (MICOS) complexes related to OMM sorting and assembly machinery (SAM) (ref. [95] and Figure 6). It is now recognized that the shape of cristae, their size, and CJs have specific patterns for different metabolic regimes and differ significantly in physiological and pathological conditions. The most important recent advances in the field are the identification of cristae-shaping proteins such as rows of ATP synthase dimers forming the cristae lamella edges, MICOS subunits, optic atrophy 1 (OPA1) isoforms, mitochondrial genome maintenance 1 (MGM1) filaments, prohibitins, and some others. Moreover, it has become clear that ultra-morphology of mitochondrial cristae reflects metabolism, redox homeostasis, and pathology, e.g., disordered cristae typically result in higher superoxide production (ref. [95] and references therein). The mobility and composition of OPA1, MICOS, and ATP synthase dimeric rows, regulated by post-translational modifications, play a decisive role in changing the morphology of cristae; nevertheless, ion fluxes across CMs, such as selective K^+^ fluxes and others, are important as well (ref. [95], see also [34,35] and Figure 6). The shape of mitochondrial cristae determines mSCs assembly of mETC and respiratory efficiency. An absence of energy substrates evokes OPA1 oligomerization and narrowing of cristae, which is needed for F_1_F_0_-ATPase assembling and for ATP-linked respiration [96]. In addition, the association between OPA1 and CI, CII, and CIII of mETC was found as well [97]. Moreover, according to available proteomics data, this protein can potentially connect to mBK(Ca) [94]. At present, it is considered that mBK(Ca) locates directly in multiprotein complexes residing in CMs, and its activity can influence cristae volume (regulation of respiration) and opening of CJs (regulation of apoptosis connected with cytochrome c release) (refs. [28,66] and references therein). Noteworthily, new data has emerged that not only mBK(Ca) [77,85] but also several other selective mitochondrial potassium channels, such as a small conductance Ca^2+^-activated K^+^ channel (mSK(Ca)) [98], the voltage-dependent KCNH6 channel [99], and also possibly mKATP (refs. [34,95] and references therein), are tightly related to different respiratory chain components, influence mSCs assembly, modulate cristae size and mitochondrial ROS formation, and regulate mitochondrial structure and function.

In the literature in the field, there is evidence that Cd^2+^ can bind with Ca^2+^ binding sites of BK(Ca) and activate the channel [36,37,100] and references therein. In this connection, it is worth recalling that BK(Ca) is a tetrameric structure composed of a pore-forming domain, BK(Ca)-α, encoded by *KCNMA1*, and two accessory domains—BK(Ca)-β and BK(Ca)-γ. Each α subunit of BK(Ca) contains three main domains: a voltage sensor domain (VSD, S0–S4) and a pore-gate domain (S5–S6), both of which are transmembrane ones, as well as a substantial internal C-terminus region, which functions as a Ca^2+^ sensor domain (S7–S10). The Ca^2+^ sensor domain consists of two non-identical regions, namely two K^+^ conductance regulator (RCK) domains 1 and 2, and RCK1 and RCK2 (Ca^2+^ bowl), containing two unique high-affinity Ca^2+^ binding sites, mediate the allosteric gating. Importantly, Ca^2+^ binding with these two separate high-affinity sites activates the channel by different mechanisms (refs. [36,37,50,100] and references therein). BK(Ca) activation is also affected by electrostatic interaction between Mg^2+^ and RCK1; RCK1 controls the sensitivity of the channel to Zn^2+^ and Cd^2+^ [37,100,101,102,103]. Notably, the differences between the Ca^2+^ binding sites of the channel were found quite a long time ago in experiments using Cd^2+^ and mutated Ca^2+^ bowl (i.e., a region with a number of Asp residues located inside RCK2 domain). It was shown that under the used conditions, BK(Ca) retained a partial sensitivity to Ca^2+^ whereas it sustained its susceptibility to Cd^2+^. As a result, it has been proposed that BK(Ca) has a second Ca^2+^ binding site that may also bind with Cd^2+^ to activate the channel [101]. Later, the other group of investigators confirmed that the Cd^2+^ sensitivity of RCK1 was due to the second Ca^2+^ binding site (Asp362/Asp367) where Asp362 had a weak influence on Ca^2+^ sensitivity [102,103]. Interestingly, RCK domains can sense several other divalent cations, namely Sr^2+^, Ba^2+^, Mn^2+^, Co^2+^, or Ni^2+^; moreover, an additional low-affinity Ca^2+^ binding site for metal ions with smaller radii such as Mn^2+^, Co^2+^, Ni^2+^, and Mg^2+^ was supposed [102,103].

Keeping this in mind, it seems quite possible that Cd^2+^ binds with Ca^2+^ binding sites of BK(Ca)s in PC12 and AS-30D cells and may affect the functioning of these channels; moreover, the mitigating effect of Pax on Cd^2+^-induced cytotoxicity observed in our experiments may be due to the antagonistic effect of this compound on Cd^2+^-promoted disturbance of these channels. It should be noted that for PC12 cells, the presence of mBK(Ca)s in these cells is well documented [76]; in the case of AS-30D cells, this issue is the subject of further studies. In addition, in our previous work [35], we suggested that the mitigating action of Pax on Cd^2+^-induced toxic damage of PC12 and AS-30D cells may be due to the ability of this compound to somehow counteract the formation of mSCs, which themselves or their components could be involved in the mPTP assembly process in the presence of the heavy metal (see below for the discussion of the relationship between mETC, mPTP, and Cd^2+^ [34]). Remarkably, these two assumptions are in good agreement with each other. Furthermore, in recent years, much information has accumulated regarding significant alterations/modifications of respiratory SCs assembly in various pathological conditions, including metabolic diseases [104]; in particular, the existence of such respiratory SCs such as CI_1_CIII_2_CIVn (respirasomes), CICIIICIV, CI_1_CIII_2_, CIII_2_CIVn, etc., is well documented (Refs. [45,104] and references therein).

In this regard, it should be reminded that in our previous experiments on isolated RLMs, it has been found that Cd^2+^ exerts discrete modes of action on SH- and Ca^2+^-dependent membrane domains, which are critical for IMM permeabilization; moreover, this heavy metal ion is able to activate opening of mPTP both in its high- and low-conductance states [24,25,26,27]. It has also been shown that rotenone (Rot) and/or stigmatellin (Stig), i.e., selective inhibitors of CI and CIII of mETC (I_Q_ and III_Q0_ sites, respectively), are often no less effective than cyclosporine A (CsA, a powerful mPTP inhibitor/”desensitizer”), dithiothreitol (DTT, a potent dithiol reducing agent), N-acetylcysteine (NAC, a GSH precursor), or ruthenium red (RR, a selective inhibitor of mitochondrial Ca^2+^-uniporter) in the suppression of Cd^2+^-induced ROS production changes, mPTP opening, and cell death. Thus, the existence of at least two distinct sets of Cd^2+^ binding sites (accessible to this heavy metal from the matrix and cytosol sides of the IMM, respectively) has been demonstrated. As recognized also, the sites, which are responsible for changes in IMM permeability, may coincide with or be vicinal to sites modulating respiration changes in the presence of Cd^2+^ and represent critical sites of mPTP formation/regulation, i.e., critical dithiols and Ca^2+^(Me^2+^) binding sites [12,13,14,24,25,26,27,30,38,39,40].

Importantly, more than 20 years ago, a hypothesis about a crucial involvement of respiratory complexes in the formation/regulation of nonselective mitochondrial pores (Belyaeva’s hypothesis, 2004) [27,105] was put forward and was subsequently supplemented, taking into account data obtained in the following years [12,13,14,34,38,39,40,41,44]. Remarkably, experiments carried out with Cd^2+^ provided the decisive basis for this theory. It has been suggested that both CI and CIII of mETC (per se or in the form of their SCs) directly and/or via their ROS generation contribute crucially to IMM permeabilization induced by Cd^2+^ and/or Ca^2+^. Some of these critical sites are believed to be located (1) in the RET pathway from Succ to NAD^+^ somewhere near the Rot binding site, I_Q_ (CI), and (2) on cyt *b* somewhere near heme b_L_ and close to the Stig binding site, III_Q0_ (CIII) [27]. It has also been proposed that CI constitutes the “P” redox-sensitive site of mPTP (i.e., sensitive to the PN redox state), whilst CIII is its “S” redox-sensitive site (i.e., sensitive to the thiol/dithiol redox state), and depending on cell type and conditions used, either one or both these complexes could be involved in triggering of mPTP assembly [27,105]. Additionally, it has been suggested that an achievement of a crucial closeness of the Ca^2+^(Cd^2+^) binding loci on CI/CIII and/or ANT facilitated by cyclophilin-D (CyP-D) due to its peptidylprolyl *cis-trans* isomerase activity becomes a trigger for conformational changes leading to mPTP formation [105].

Furthermore, our results obtained at the cellular level using AS-30D and PC12 cell lines imply that the increased ROS generation alone is not sufficient to induce either necrotic and/or apoptotic death by heavy metal ions such as Cd^2+^, Hg^2+^, and Cu^2+^, so there must be additional factor(s) contributing to the cytotoxic effects of heavy metals, and a partial blockade of mETC appears to be one of the most important among them [12,14]. In addition, the further experiments provided evidence indicating an effect of Cd^2+^ on the II_Q_ region of mETC, i.e., near the ubiquinone (Q) binding site of CII [41]. On the whole, the findings obtained by us in recent years indicate that not only an increase in ROS production by mETC but also conformational changes in the “Q-zone” (I_Q_, II_Q_, and III_Q0_) of mETC are crucial for Cd^2+^ and/or Ca^2+^-induced mitochondrial dysfunction that is involved in cell death and pathological conditions of different types [41]. Finally, based on our own findings and data existing in the literature in the field, it has been postulated that Cd^2+^, or Ca^2+^ plus P_i_, or Ca^2+^ (in the presence of different thiol reagents or pro-oxidants), or high Ca^2+^ as well as ROS produced by them are very likely signals and/or tools for the critical conformational changes leading to disassembly/disintegration of mitochondrial respirasome/ATP synthasome SCs with subsequent incorporation of their components in a specific manner (depending on the conditions and type of cells used) into mPTP assembly, causing the opening of mitochondrial nonselective pores [34,41,44] (see Figure 6).

It is important to add that in the previous works using isolated RLMs, we found that Cd^2+^ was able to induce both CsA-sensitive (at low [Cd^2+^]) and CsA-nonsensitive (at high [Cd^2+^]) mitochondrial membrane permeabilization and respiratory rate changes, which responded differently to several other mPTP modulators such as DTT, EGTA (ethylenebis(oxyethylene-nitrilo)]tetraacetic acid), RR, selective mETC inhibitors, and some others [13,25,26,27,30,40]. Later, another group of researchers conducted independent experiments on isolated RLMs using high and low Cd^2+^ and not only confirmed our findings but also supplemented them in some very important aspects [106]. In particular, these researchers showed that Cd^2+^ induced significant changes in the mitochondrial membrane fluidity, and these changes were specifically caused by its action on protein sites/domains. In the case of Ca^2+^, the addition of low Cd^2+^ induced the increase of mitochondrial membrane fluidity together with mitochondrial swelling and ΔΨ_mito_ decrease, which were inhibited by CsA, indicating that low Cd^2+^ led to the assembly of mPTP acting via the Ca^2+^-dependent domain. In turn, DTT and MBM^+^ (“S” site protective agents) partially prevented the changes in membrane fluidity induced by low Cd^2+^; DTT significantly decreased the swelling but was not effective in blocking of the ΔΨ_mito_ loss [106]. These results confirmed that both the “S” and Ca^2+^-triggering sites were involved in the low Cd^2+^-induced mPTP opening. On the contrary, using of high Cd^2+^, the decrease in membrane fluidity was observed, and the addition of chelating agent EGTA led to the restoration of the membrane fluidity. In addition, DTT strongly inhibited the decrease of membrane fluidity in the presence of high Cd^2+^ whilst MBM^+^ and CsA failed to prevent these changes; DTT inhibited the swelling and collapse of ΔΨ_mito_ at high Cd^2+^ while CsA was ineffective. In the opinion of these authors, the results indicate that the Ca^2+^-triggering site does not play a substantial role in the toxic action of high doses of Cd^2+^, and some other binding model has become the main one. These researchers also showed that low Cd^2+^ caused a partial aggregation of membrane proteins, whereas high doses of Cd^2+^ induced the potent aggregation; nevertheless, both of these injuries were prevented by DTT [106].

In this connection, it should be noted that new important information has recently appeared related to the continuation of research in the above direction, as a result of which Cd^2+^ binding to IMM phospholipid cardiolipin (CL) has been revealed, which disrupts respirasome assembly and redox balance via mitochondrial membrane rigidification [46]. Specifically, on rat kidney cortex (rKC) mitoplasts/mitochondria and human proximal convoluted tubule (HPCT) cells, the following results were obtained. In HPCT cells, a 30% loss of CICIII_2_CIV_n_ SCs formation after treatment with Cd^2+^ for 6 h was shown, which was reversed by the CL-binding drug MTP-131/SS-31/elamipretide. The Cd^2+^-induced ROS rise was also decreased (25%) in the presence of this compound in the cells. In rKC mitochondria, MTP-131 reduced Cd^2+^-induced H_2_O_2_ increase (30%) and cytochrome c release (25%) but had no effect on their osmotic swelling. Heterologous CRLS1 expression reversed the Cd^2+^-produced cytotoxicity measured by MTT assay, Cd^2+^-induced ROS increase, and the mitochondrial membrane rigidification caused by this heavy metal in HPCT cells. Overall, the novel mechanism of Cd^2+^-induced cytotoxicity has been identified by these researchers in which Cd-CL interactions cause IMM rigidification and thereby disrupt the proper SCs assembly and increase ROS levels [46].

Importantly, one more interesting work cannot be overlooked [45]. Using respirasome and free CI isolated from *U. maydis* mitochondria and 7 metals/metalloids of toxicological relevance, namely As^3+^, Fe^3+^, Zn^2+^, Cd^2+^, Cr^6+^, Cu^2+^, and Hg^2+^, it has been studied their action on the NADH:DBQ oxidoreductase activity and ROS generation. As a result, free CI was found to be more resistant to the inactivation by heavy metals than the respirasome. Furthermore, the respirasome inactivation by the metal/metalloids used did not significantly increase ROS formation, whereas free CI was more sensitive to electron leak and ROS production in their presence [45]. In particular, heavy metals inhibited dose-dependently the respirasome activity, and Hg^2+^ was the most toxic among them, whilst Cd^2+^—the least toxic (IC_50_ = 150 µM). In turn, free CI was more resistant to heavy metals than the respirasome, except for Cu^2+^ [45]. In the case of Cd^2+^, the IC_50_ value (for free CI) was 2 times greater than the IC_50_ value for the respirasome, while Hg^2+^ did not affect free CI activity, even at 1 mM. Fitting the data to the Hill equation revealed the significant difference in the number of ions binding to the respirasome or free CI. In the respirasome, Cd^2+^, Hg^2+^, and Cu^2+^ showed the cooperative binding, but in the case of other ions used, the kinetics were hyperbolic. All of this means that the interaction of heavy metals with the respirasome and free CI is completely different [45]. As found also by these researchers, the inhibition of respirasomal NADH:DBQ oxidoreductase activity by heavy metals increased the H_2_O_2_ production rate, with the maximum observed at IC_75_ for Cu^2+^ (close to 1% of the total electron flow), whilst for free CI (about 24% of the total electron flow during NADH oxidation). Of note, Cu^2+^ was the greatest ROS inducer for both respirasome and free CI, whereas even 1 mM Hg^2+^ did not significantly increase ROS in free CI (in agreement with the absence of inactivation of free CI by Hg^2+^) [45]. As was shown also by these authors, if the inhibition of the respirasome and free CI enhanced, the electron leakage increased as well; nevertheless, the ROS production was found to be small at IC_25_ or IC_50_, while at 75% inhibition, there was a burst of electron leaks, indicating the most aggressive damage of the NADH dehydrogenase function by the heavy metals. Notably, the inhibition of the respirasome by Cd^2+^ did not induce the surge in electron leakage. This important result indicates that the inactivation of NADH:DBQ oxidoreductase activity of the respirasome by Cd^2+^ occurs at sites that are not associated with ROS production [45]. Remarkably, these observations agree well with our findings obtained previously on such divalent heavy metal ions as Cd^2+^, Hg^2+^, and Cu^2+^, which became the basis for the aforementioned theory about the critical involvement of respiratory complexes in Cd^2+^ or/and Ca^2+^-induced mPTP pore opening (see above the discussion on Belyaeva’s hypothesis, 2004) [12,13,14,27,34,38,39,40,41,44,105].

In addition, it is worthy to recall that quite a long time ago, it has been found that Cd^2+^ can bind not only with F_1_F_0_-ATPase per se (a catalytic site of its β-subunit is now considered the most likely candidates for the role of the Ca^2+^-trigger site of mPTP [29]) but interfere in the interaction between ATP synthase and its regulatory factors, namely ATPase Inhibitory Factor 1, IF_1_ (i.e., a natural protein inhibitor of F_1_F_0_-ATPase), ATP synthase Coupling Factor B, and FB (or ATP synthase subunit s, ATP5S, or distal membrane-arm assembly complex 2-like protein, DMAC2L), the latter of which (DMAC2 or ATP5SL) is also necessary for assembly of the distal portion of the membrane arm in human mitochondrial respiratory CI. Of note, both of these regulatory factors participate in the remodeling of cristae structure and in the regulation of mitochondrial morphology, and in this way Cd^2+^ can affect the mitochondrial ultrastructure and function as well. Not only F_1_F_0_-ATPase but also ANT and P_i_C, which are all components of mitochondrial ATP synthasome SCs (Figure 6), are well-known targets for this heavy metal (for more details, see [34] and references therein).

Thus, there is a tight relationship (structural, functional, and/or regulatory) between mETC, mPTP, and mBK(Ca), and Cd^2+^ may interfere with these processes, having both a negative and positive impact.

In this regard, it is important to mention numerous publications that have appeared in recent years concerning the mutual influence of Cd^2+^ and flavonoid quercetin on each other’s effects; in particular, this compound can both protect against and enhance the toxic effects of Cd^2+^ [107,108,109,110,111,112,113,114]. Quercetin, as it is known, is a naturally occurring flavonoid that is widely found in plants used by humans as food. It has been shown to have powerful antioxidant, anti-inflammatory, anti-aging, antibacterial, and anticancer properties [115]. Quercetin has been found to have multiple targets within the cell, particularly in the mitochondria. As revealed, this compound is able to inhibit (at low concentrations) or activate (at high concentrations) mPTP [116]; it inhibits F_1_F_0_-ATPase itself, namely ATPase activity of CV of mETC [117]. In addition, CI of mETC and cyt C are molecular targets of quercetin that inhibit hydrogen peroxide production by mitochondria [118]. Interestingly, the extent of CI inhibition by quercetin was found to be dependent on the concentration of CoQ in the medium, suggesting the competition between the flavonoid and CoQ for close binding sites in the complex [118]. It has also been reported that quercetin is able to protect CI from inhibition by different toxic agents, such as various non-steroidal anti-inflammatory drugs, and this protection is accomplished via a CoQ-like action [119].

More intriguingly, it has been recently demonstrated that one of the quercetin targets is mBK(Ca) itself [120]; furthermore, it is an activator of this channel [66,120]. Not long ago, it was also shown that quercetin abolishes the Pax inhibition of mBK(Ca) [121]. As is well-known, Pax is considered to be a canonical BK(Ca) inhibitor and has been found to bind within BK(Ca) pore [122,123]. In Szewczyk’s lab, using the patch-clamp method, it has been revealed that quercetin prevents Pax from blocking the mBK(Ca), indicating that both chemicals compete for an overlapping binding site. Furthermore, with the help of a quercetin analog, isorhamnetin, which doesn’t affect mBK(Ca) activity, these researchers showed that the subsequent Pax application resulted in a full block of the mBK(Ca), confirming that isorhamnetin didn’t interrupt the Pax binding. Additionally, in molecular docking studies conducted by these researchers, it has been found that Pax and quercetin occupy a common binding site within the BK(Ca) pore, and activation of mBK(Ca) by quercetin prevents Pax from binding [121]. These data are in good agreement with the observations obtained in the present study, which indicate the significant mutual influence of BK(Ca) modulators and Cd^2+^ on the effects of each other, as well as the potential ability of Cd^2+^ to interfere in the BK(Ca)s functioning.

It is necessary to add that there are other opinion(s) concerning the mechanism(s) of the protective effect of Pax against toxic insults of various types, excluding the participation of mBK(Ca) per se. As shown by Kulawiak and Szewczyk (2012) [58], Pax protects against glutamate toxicity in neuronal HT22 cells; nevertheless, other BK(Ca) inhibitors, namely iberiotoxin and charybdotoxin, are not cytoprotective when applied. Moreover, a structural analog of Pax, which doesn’t block BK(Ca), paxillinol exhibited an ameliorative effect on the glutamate-induced cytotoxicity as well [58]. Pax did not reduce intracellular ROS production and did not restore GSH levels in cells exposed to glutamate. As a result, these researchers concluded that the mitigating action of Pax was not related to mBK(Ca) activity or an influence on oxidative stress [58]. In the literature in the field, there is evidence on several other cellular targets of Pax [57]; for example, this compound can inhibit inositol triphosphate receptor, IP_3_R [124], and sarco/endoplasmic reticulum Ca^2+^ ATPase [125], indicating its possible modulation of Ca^2+^ homeostasis. It is likely that there are other potential cellular targets for Pax that we do not yet know about.

In conclusion, it is worthy to note that the results obtained in the present and our previous studies on this topic [35,48,49] suggest that Pax attenuates Cd^2+^-induced cell injury not only via its effects on bioenergetic function and ROS formation, but also that BK(Ca)s may participate in some way in the mechanisms of its action. Our observations indicate the differential responses of the two rat cell lines used in our experiments to the addition of Pax. At the moment, however, the reasons and physiological implications of this differential cellular response to Cd^2+^-induced toxic action in the presence of Pax are not entirely clear and require further clarification. One possible explanation for this difference could be the fact that in PC12 cells, BK(Ca)s are active from the start, whereas in AS-30D cells, active BK(Ca)s are absent or present in insufficient quantities; however, this requires careful investigation. In addition, the protective effect of Pax may be due to the induction of some intracellular signaling pathways critical for cell viability, in which mETC (through changes in its activity or its structural and/or conformational changes) and/or ROS produced by the respiratory chain, as well as BK(Ca)s, play an important role. Remarkably, in recent years, a large number of studies have been conducted aimed at elucidating the cellular signaling pathways involved in the mechanisms of the cytotoxic action of Cd^2+^ (refs. [11,19,22,126,127,128] and references therein), which can certainly help in our further research.

## 4. Materials and Methods

### 4.1. Chemicals

NS004, NS1619, paxilline, CdCl_2_, oligomycin, FCCP, CCCP were from Sigma-Aldrich Company (Saint Louis, MO, USA). PI, JC-1 and DCFH_2_-DA were obtained from Molecular Probes (Eugene, OR, USA). All cell culture supplies were products of GIBCO BRL (Grand Island, NY, USA) or Biolot (St. Petersburg, Russia). All other reagents were of the highest purity grade commercially available.

### 4.2. PC12 Cells

The rat pheochromocytoma PC12 cell culture was maintained in a CO_2_ incubator in an atmosphere containing 5% CO_2_ at 37 °C, as previously described [14,35]. As an incubation medium, we used DMEM with L-glutamine together with 25 U/mL of streptomycin, 25 µg/mL of penicillin, 10% fetal calf serum, and 5% horse blood serum (Biolot, St. Petersburg, Russia) which was renewed every two days. In some cases, the DMEM with L-glutamine and the antibiotic medium without serum was applied. The remaining details of the experimental procedure used are given in legends in the corresponding tables and figures.

We used the same design of experiments as earlier described [12,35,49]. In particular, the cells of both rat culture lines (PC12 or AS-30D) were pre-incubated, respectively, in the DMEM (Biolot, St. Petersburg, Russia) or the RPMI-1640 (GIBCO BRL, Grand Island, NY, USA) media without or with the corresponding BK(Ca) modulators for 30 min in 6-, 12-, 24-, or 48-well plates or in Petri dishes at 37 °C. Typically, NS004 and NS1619 were used at a concentration of 10 µM; however, in some cases, we used higher concentrations (30 and 100 µM) of these BK(Ca) openers. Pax was routinely applied at a concentration of 1 µM. Further, the respective concentration of Cd^2+^, where appropriate, namely 10, 30, 50, 100, or 500 µM, was added in each well or Petri dish. CdCl_2_ was dissolved in bi-distilled water to prepare a 10 mM stock solution. Before the experiments, Cd^2+^ from this stock solution was diluted with the corresponding medium or phosphate buffered saline (PBS) to the desired concentrations.

To evaluate the viability of PC12 cells, the LDH release assay as previously used [14]. Shortly, PC12 cells were seeded into 24-well plates (in a concentration of 2.5 × 10^5^ cells in each well) and after 3, 5, 24, or 48 h of incubation, the cell viability was estimated by monitoring of cellular LDH spectrophotometrically (for more details, see [14,35]). The percentage of LDH released was evaluated as a percent of enzyme activity in an incubation medium to the total LDH activity in a sample. One hundred percent of LDH activity in an incubation medium corresponds to the absence of viable cells. The cell viability was expressed as a percentage of the corresponding untreated control.

Cellular respiration was measured polarographically at 37 °C with the help of a Clark oxygen electrode (YSI, Yellow Springs, OH) in a thermostatic water-jacketed chamber with magnetic stirring. PC12 or AS-30D cells were incubated in 10 mL of the corresponding complete (DMEM or RPMI-1640) medium (with serum) in Petri dishes for 3, 5, 24, or 48 h with various concentrations of Cd^2+^ and/or the corresponding BK(Ca) modulators, then collected by centrifugation and transferred to the corresponding (DMEM or RPMI-1640) medium without serum or into PBS supplemented with 5 mM glutamine and 5 mM pyruvate. The number of cells in the respiration vessel was 10^7^. The values of cellular OCR in several energetic states for cells exposed to the respective BK(Ca) modulators were determined. Briefly, the following cellular respiratory rates were measured in each experiment: (i) in a steady state (basal respiration), (ii) after addition of oligo (resting respiration, i.e., in state 4o, St4_o_ rate), and (iii) after the following supplement of one of artificial protonophores such as FCCP or CCCP (i.e., maximal or fully uncoupled respiration in state 3u, St3_u_ rate) for PC12 and AS-30D cells, respectively. Oligo and FCCP/CCCP were used in concentrations of 5 µg/mL and 1 µM, correspondingly. OCR under used conditions was counted as a percentage of the maximal (fully uncoupled) respiration rate of the respective control cells, i.e., as a percentage of V_FCCP_(St3_u_) of the control—in the case of PC12 cells and as a percentage of V_CCCP_(St3_u_) of the control—in the case of AS-30D cells.

The ΔΨ_mito_ changes in PC12 cells (as well as in AS-30D cells; see Section 4.3) were determined using JC-1 dye (Molecular Probes) [129,130]. Noteworthily, a lipophilic cationic probe, JC-1, accumulates in mitochondria with high ΔΨ_mito_ and, due to molecular stacking, changes its fluorescence from green to red. In PC12 cells, all incubations were conducted in the DMEM medium as described above; then, after treatment with the appropriate agents, they were washed with Hank’s balanced salt solution (HBSS); after that, they were incubated with 2 µM JC-1 in HBSS for 15 min in the dark at 37 °C. After washing the cells, fluorescence was immediately determined using a CLARIOstar Plus plate reader (BMG Labtech, Ortenberg, Germany). Fluorescence was measured at Ex/Em: 535 nm/595 nm for JC-1 aggregates and at Ex/Em: 485 nm/535 nm for monomers. FCCP was added to the wells as a positive control. The red/green fluorescence ratio of the dye in mitochondria can be considered as a direct assessment of the state of the mitochondria polarization; namely, the higher the red-to-green ratio of the fluorescent marker, the more aggregates are formed. A reduction of the red-to-green fluorescence intensity ratio is connected with mitochondrial depolarization.

An intracellular ROS production of PC12 cells (and AS-30D cells, see Section 4.3) was determined using the fluorescent dye DCFH_2_-DA as previously [12,14,35]. PC12 cells were seeded to 24-well plates to estimate the ROS accumulation. The experiments were conducted in the DMEM containing L-glutamine and started 24 h after the addition of the cells to the plates. The cells were incubated with 10 µM of DCFH_2_-DA for 30 min in the dark at 37 °C. The plates were washed 2 times with HBSS. After that, the fluorescence of 2′,7′-dichlorofluorescein, a reaction product of ROS with a deacylated dye, was measured on a Fluoroscan Ascent FL plate fluorimeter (Thermo Fisher Scientific, Vantaa, Finland) at excitation and emission wavelengths of 485 nm and 538 nm, respectively. The ROS content was expressed in arbitrary units (a.u.).

### 4.3. AS-30D Cells

The culture of rat ascites hepatoma AS-30D cells, kindly provided by Dr. Antonio Villalobo (Institute for Biomedical Research, National Research Council and Autonomous University of Madrid, Spain), was maintained in the same way as the PC12 cell culture, i.e., in a CO_2_ incubator at 37 °C in an atmosphere containing 5% CO_2_. As an incubation medium, RPMI-1640 medium (with 40 μg/mL gentamycin, 10% fetal calf serum, and 2 mM L-glutamine) as previously used [12,38]. Of note, these cells are easily cultivated in vitro and characterized not only by an intense glycolysis but also by high rates of respiration and oxidative phosphorylation. The design of experiments and the procedure of determination of cellular respiration were described in detail in Section 4.2.

To estimate the viability of the AS-30D cells, we used the TB exclusion test as earlier [12,38]. For this, AS-30D cells were seeded at a density of 0.5 × 10^6^ cells/mL and then used after being cultured overnight. Cell viability was expressed as the percentage of cells that did not accumulate the dye, i.e., the TB-negative cells. The quantity of apoptotic cells was determined by flow cytometry as a sub-G_1_ fraction after PI staining of cells according to the procedure of Nicoletti et al. (1991) [131]. Flow cytometry was conducted using a FACS Calibur instrument (FL-2 channel) with Cell-Quest 3.0 software (Becton Dickinson, San Jose, CA, USA). DNA content frequency histograms were obtained during experiments, and this gave us an opportunity to discriminate between cells with normal (diploid) DNA content and those forming a broad hypodiploid DNA peak (the sub-G_1_ population). About 10^4^ cells were used for each run. More details of the experimental procedure are presented in legends to the respective figures.

To evaluate changes in ΔΨ_mito_ of AS-30D cells, we stained the cells with JC-1, as mentioned above in Section 4.2. The ΔΨ_mito_ changes were monitored using channels FL-1 (green) and FL-2 (orange-red) of the same flow cytometer and the same software (see above) as described previously [12,38]; for more details, see [129] and legends to the corresponding figures.

To measure intracellular ROS formation in AS-30D cells, the same probe was used as for PC12 cells (see Section 4.2). The cells were incubated with 20 μM of DCFH_2_-DA at 37 °C for 30 min and later analyzed by flow cytometry using a green light-sensitive photomultiplier (FL-1). The ROS production was estimated as a geometrical mean of the total green fluorescence of DCF as previously [12,38]. All measurements were conducted using the same flow cytometer and the same software (see above). For each run 10,000 cells were applied. For more details, see [129] and the legends to the respective figures.

### 4.4. Statistical Analysis

The results presented are an average (mean values ± SEM) or a representation of a series of at least three independent experiments, unless otherwise indicated. As in our previous publications, the statistical significance was analyzed using, where appropriate, one-way or two-way ANOVA or Student’s *t*-test with *p* < 0.05 assumed as the significance threshold.

## 5. Conclusions and Perspectives

In this study, we investigated the effects of two synthetic BK(Ca) activators, NS004 and NS1619, on the neuro- and hepatotoxicity of Cd^2+^, and the effects of the BK(Ca) inhibitor, Pax, on their actions in the absence and presence of this heavy metal, using two rat cell lines, PC12 and AS-30D. Specifically, we have studied the cell viability, apoptosis/necrosis, respiration, ΔΨ_mito_, and intracellular ROS production with the combined use of these BK(Ca) modulators and Cd^2+^.

We identified the dual nature of the NS004/NS1619 effects (short-term protection versus long-term damage worsening) that was crucial for the interpretation of our data. Additionally, we have found that the significant changes in the intracellular ROS formation induced by NS004/NS1619 in the absence or in the presence of Pax and/or Cd^2+^ are the early cellular effects of the BK(Ca) activators used. Furthermore, mETC and changes in intracellular ROS production are among the key factors in the molecular mechanisms of action of the studied BK(Ca) modulators. Importantly, our results also suggest that BK(Ca)s may somehow be involved in the observed cellular events. It is worth noting that this work is a logical continuation of our previous research on this topic, and we hope that it will contribute to the understanding of the complex interplay between ion channels, mitochondrial function, and heavy metal toxicity. Moreover, we attempted to integrate our results into the current paradigm by discussing the relationship of BK(Ca)s with mETC, mPTP, and cristae structure, as well as with several other cellular/mitochondrial targets (Figure 6).

We plan to continue studying this issue in more detail in the future. For example, one of the possible explanations for the observed effects of NS004/NS1619/Pax could be that their action is mediated not (or only partially) by direct channel opening/closing but also by modulating its expression, in addition to influencing mETC and intracellular ROS levels. It should be noted once again that, at this point, it is only possible to note that the involvement of BK(Ca) channels in the mechanisms being studied is very likely due to the limitations of the pharmacological approach used in this work and the existence of multiple off-target effects of the BK(Ca) modulators (see pp. 3, 19, 20, 26). Direct confirmation of the specific role of BK(Ca) channels in this process will require data on knockdown of the KCNMA1 gene; data on BK(Ca) channel mRNA or protein expression, particularly of their mitochondrial isoform (with the VEDEC splice variant), will be very useful. Furthermore, it is necessary to confirm the presence of mitoBK(Ca) in the cell lines used in this study and determine how its levels are affected by Cd^2+^ and the modulators. Direct experimental data, such as recording mitoBK(Ca) activity, are also needed.

Finally, it is worthy to emphasize that both Cd^2+^ and BK(Ca) modulators are excellent tools for elucidating the molecular mechanisms of the relationship between mBK(Ca), mETC, and mPTP and the influence of ubiquitous environmental toxicants such as heavy metals on them. One of the promising directions for our future research is a careful exploration of the possibility to activate/inhibit the mBKCa by Cd^2+^ and the potential ability of Pax/NS004/NS1619 to influence this (in the absence and in the presence of various mETC and mPTP modulators). Additionally, it is very important to make a comparison with the action of other heavy metal ions, namely Zn^2+^, Cu^2+^, Hg^2+^, and some others under the same conditions. A study of signaling pathways involved in the effects observed in the presence of Cd^2+^ and/or mBK(Ca)/mK(ATP)/mPTP modulators shown in the current and our previous works is also relevant. Furthermore, we are sure that there are many interesting discoveries to be made in this area in the near future.

## Figures and Tables

**Table 1:** Estimation of respiratory rates in different energetic states (as a percentage of V_FCCP_(St3_u_) of the control) in PC12 cells exposed to NS004 or NS1619 for different incubation times.

Time	Respiration Status	Control	NS004	NS1619
3 h	Steady state	35 ± 5	41 ± 3	43 ± 3
	+ Oligo (St4_o_)	16 ± 2	20 ± 1 *	23 ± 2 *
	+ FCCP (St3_u_)	100	100	102 ± 2
5 h	Steady state	40 ± 4	38 ± 2	33 ± 3
	+ Oligo (St4_o_)	19 ± 1	14 ± 4	19 ± 1
	+ FCCP (St3_u_)	100	80 ± 3 *	92 ± 5 *
24 h	Steady state	53 ± 7	39 ± 9	52 ± 14
	+ Oligo (St4_o_)	22 ± 3	21 ± 5	23 ± 8
	+ FCCP (St3_u_)	100	82 ± 4 *	83 ± 4 *

The cellular OCR was counted as a percentage of the maximal (fully uncoupled) respiration rate of the respective control cells, i.e., as a percentage of V(St3_u_) of the control. To obtain the maximal respiratory rates of PC12 cells, a chemical protonophore, FCCP, was added (for more details, see Section 4.2). The data is shown as mean ± SEM for 3–5 independent experiments. Statistical significance: * *p* < 0.05 with respect to the corresponding control. The concentrations of the BK(Ca) activators, NS004 and NS1619, were 10 µM.

**Table 2:** Changes in mitochondrial membrane potential (ΔΨ_mito_) of PC12 cells exposed to different concentrations of NS004/NS1619 and/or Cd^2+^ for different incubation times (expressed as a percentage of the control).

Time	[Cd], µM	(None)	NS004 (10 µM)	NS1619 (10 µM)	NS004 (30 µM)	NS1619 (30 µM)
3 h	0	100	97 ± 3	95 ± 5	92 ± 8	93 ± 7
	10	97 ± 4	n.d.	n.d.	n.d.	n.d.
	30	95 ± 5	n.d.	n.d.	n.d.	n.d.
	50	94 ± 6	94 ± 7	98 ± 10	98 ± 8	90 ± 11
	100	93 ± 7	93 ± 9	95 ± 5	94 ± 14	91 ± 16
	500	31 ± 5 *	30 ± 6 *	35 ± 11 *	35 ± 7 *	30 ± 6 *
5 h	0	100	93 ± 7	99 ± 6	94 ± 6	94 ± 5
	50	109 ± 11	107 ± 8	104 ± 5	101 ± 9	96 ± 5
	100	114 ± 15	99 ± 5	95 ± 5	86 ± 7 *^§^	85 ± 6 *^§^
16 h	0	100	100 ± 3	106 ± 6	95 ± 5	97 ± 8
	10	97 ± 4	n.d.	n.d.	n.d.	n.d.
	30	95 ± 5	n.d.	n.d.	n.d.	n.d.
	50	59 ± 7 *	69 ± 11 *	67 ± 9 *	61 ± 6 *	62 ± 9 *
	100	25 ± 2 *	n.d.	n.d.	n.d.	n.d.
24 h	0	100	98 ± 4	99 ± 5	85 ± 5 *	92 ± 1 *
	10	105 ± 5	n.d.	n.d.	n.d.	n.d.
	30	89 ± 5 *	n.d.	n.d.	n.d.	n.d.
	50	47 ± 6 *	54 ± 4 *	51 ± 6 *	26 ± 1 *^§^	17 ± 1 *^§^
	100	16 ± 4 *	17 ± 7 *	19 ± 7 *	13 ± 5 *	13 ± 4 *

The ΔΨ_mito_ values (red/green: high ΔΨ_mito_/low ΔΨ_mito_, JC-1 assay method, see Section 4.2) in PC12 cells are shown as a percentage of the respective untreated control. The results are presented as mean ± SEM for 3–8 independent experiments (* *p* < 0.05 compared to the corresponding untreated control, ^§^ *p* < 0.05 compared to Cd alone), n.d.—not determined.

**Table 3:** Intracellular ROS production in PC12 cells exposed to NS004/NS1619 and/or Pax for different times of incubation in the absence and in the presence of Cd^2+^ (expressed in arbitrary units, a.u.).

Time	Additions	ROS Production (a.u.)
**30 min**	None	3.0913 ± 0.0743
	NS1619	3.2022 ± 0.2396
	NS1619 + Pax	2.798 ± 0.66
	NS004	3.0538 ± 0.1218
	NS004 + Pax	2.868 ± 0.154
	Pax	3.2765 ± 0.1765
	Cd	3.649 ± 0.1388 *
	Cd + Pax	3.3155 ± 0.1792 ^§^
	Cd + NS1619	3.3842 ± 0.2488
	Cd + NS1619 + Pax	2.684 ± 0.1004 *^§†^
	Cd + NS004	3.4575 ± 0.4532
	Cd + NS004 + Pax	2.702 ± 0.2435 *^§†^
**3 h**	None	6.355 ± 0.347
	NS1619	9.4865 ± 0.6135 *
	NS1619 + Pax	7.052 ± 0.268 ^#^
	Pax	6.445 ± 0.345
	Cd	9.969 ± 0.602 *
	Cd + Pax	8.6005 ± 0.5005 *^§^
	Cd + NS1619	11.363 ± 0.063 *^§#^
	Cd + NS1619 + Pax	8.412 ± 0.597 *^§$^

PC12 cells were incubated for 30 min or 3 h in the presence of the respective BK(Ca) modulators and/or the heavy metal. The intracellular ROS formation was measured with the help of the DCF assay method (see Section 4.2). The results are mean ± SEM (for n = 3–4 independent experiments). Statistical significance: * *p* < 0.05 compared to the corresponding untreated control; ^§^ *p* < 0.05 compared to Cd alone; ^†^ *p* < 0.05 compared to Cd + Pax; ^#^ *p* < 0.05 compared to NS1619 alone; ^$^ *p* < 0.05 compared to Cd + NS1619. The concentrations of the BK(Ca) modulators used were 10 µM for NS004/NS1619, 1 µM for Pax, and 100 µM for [Cd].

**Table 4:** Intracellular ROS production in AS-30D cells exposed to NS004/NS1619 and/or Pax for different times of incubation in the absence and in the presence of Cd^2+^ (expressed as a percentage of the respective control).

Time	Additions	ROS Production (%)
**50 min**	None	100
	NS004	68 ± 5 *
	NS1619	60 ± 3 *
	Pax	120 ± 22
	Cd	121 ± 3 *
	Cd + NS004	84 ± 10 ^§^
	Cd + NS1619	111 ± 5 ^§^
	Cd + Pax	120 ± 9 *
**3 h**	None	100
	NS004	149 ± 15 *
	NS1619	151 ± 15 *
	Pax	165 ± 29 *
	Cd	129 ± 18 *
	Cd + NS004	146 ± 10 *
	Cd + NS1619	163 ± 16 *
	Cd + Pax	147 ± 20 *
**24 h**	None	100
	NS004	118 ± 25
	NS1619	116 ± 3 *
	Pax	149 ± 20 *
	Cd	105 ± 21

AS-30D cells were incubated for 50 min, 3 h, or 24 h in the presence of the respective BK(Ca) modulators and/or Cd^2+^. The intracellular ROS formation was measured with the help of the DCF assay method (see Section 4.3). The concentrations of the BK(Ca) modulators used were 10 µM for NS004/NS1619 and 1 µM for Pax. [Cd^2+^] = 100 µM. The results are mean ± SEM (for n = 3–4 independent experiments). Statistical significance: * *p* < 0.05 compared to the corresponding untreated control; ^§^ *p* < 0.05 compared to Cd alone.

## Data Availability

The original contributions presented in this study are included in the article. Further inquiries can be directed to the corresponding author.

## Supplementary Materials

The following supporting information can be downloaded at: https://www.mdpi.com/article/10.3390/ijms262010048/s1.

## Abbreviations

The following abbreviations are used in this manuscript:

BK(Ca)	Ca^2+^-activated big-conductance potassium channel
pBK(Ca)	plasmatic BK(Ca)
mBK(Ca)	mitochondrial BK(Ca)
ROS	reactive oxygen species
RLM	rat liver mitochondria
mPTP	mitochondrial permeability transition pore
K(ATP)	ATP-sensitive potassium channel
mK(ATP)	mitochondrial K(ATP)
mETC	mitochondrial electron transport chain
IMM	inner mitochondrial membrane
Pax	paxilline
NS004	5-trifluoromethyl-1-(5-chloro-2-hydroxyphenyl)-1,3-dihydro-2H-benzimidazole-2-one
NS1619	1,3-dihydro-1- [2-hydroxy-5-(trifluoromethyl) phenyl]-5-(trifluoromethyl)-2H-benzimidazole-2-one
ΔΨ_mito_	mitochondrial membrane potential
LDH	lactate dehydrogenase
OCR	oxygen consumption rate
FCCP	carbonyl cyanide *p*-trifluoromethoxyphenylhydrazone
CCCP	carbonyl cyanide 3-chlorophenylhydrazone
oligo	oligomycin
CI-CV	respiratory complexes I, II, III, IV, or V of mETC
F_1_F_0_-TPase	ATP synthase or CV of mETC
JC-1	5,5′,6,6′-tetrachloro-1,1′,3,3′- tetraethylbenzimidazolcarbocyanine iodide
DCFH_2_-DA	2′,7′-dichlorodihydrofluorescein diacetate
TB	trypan blue
PI	propidium iodide
RET	reverse electron transport
FET	forward electron transport
mSCs	mitochondrial supercomplexes
OXPHOS	oxidative phosphorylation system
ANT	adenine nucleotide translocase
P_i_C	mitochondrial phosphate carrier
VDAC	voltage-dependent anion channel
IBM	inner boundary membrane
OMM	outer mitochondrial membrane
CMs	crista membranes
CJs	crista junctions
MICOS	mitochondrial cristae organizing system
SAM	OMM sorting and assembly machinery
OPA1	optic atrophy 1 protein
VSD	voltage sensor domain
RCK1/2	K^+^ conductance regulator domains 1 and 2
Rot	rotenone
Stig	stigmatellin
CsA	cyclosporine A
DTT	dithiothreitol
RR	ruthenium red
CyP-D	cyclophilin D
CoQ, or Q	ubiquinone
EGTA	ethylenebis(oxyethylene-nitrilo)]tetraacetic acid
CL	cardiolipin
IF_1_	ATPase inhibitory factor 1
FB	ATP synthase coupling factor
PBS	phosphate-buffered saline
HBSS	Hank’s balanced salt solution
