# Supplementary figures and images for: Application of Modulators of Ca2+-Activated Big-Conductance Potassium Channels Against Cd2+-Induced Cytotoxicity: A Study on Two Rat Cell Lines, PC12 and AS-30D
Source: Int J Mol Sci. 2025 Oct 15;26(20):10048. doi: 10.3390/ijms262010048 (PMC12564761; doi:10.3390/ijms262010048)

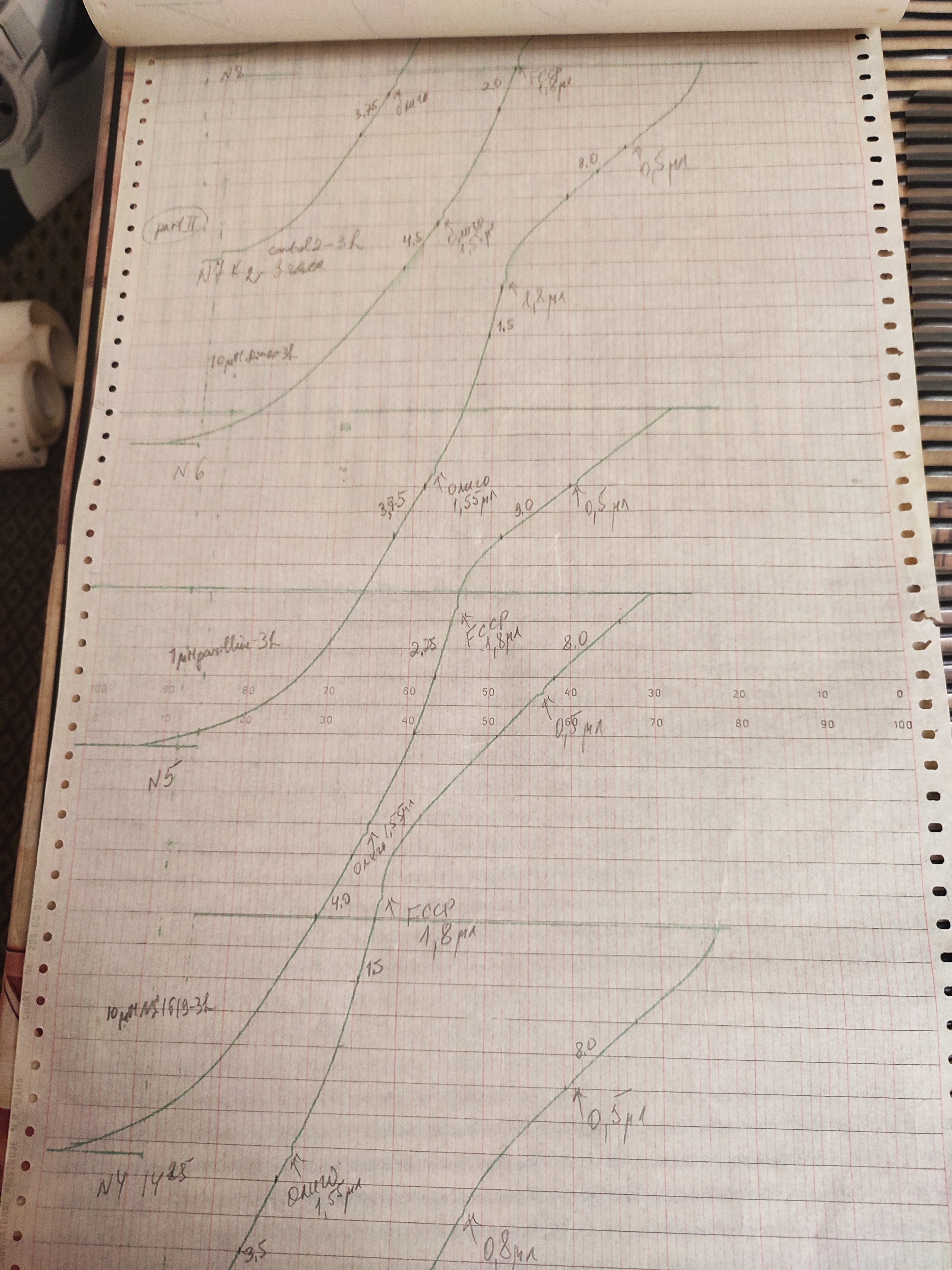

Supplement: Supplementary file 1 [file ijms-26-10048-s001.zip › FigS2-3h-10NS1619-1Pax-PC12cells.jpg]

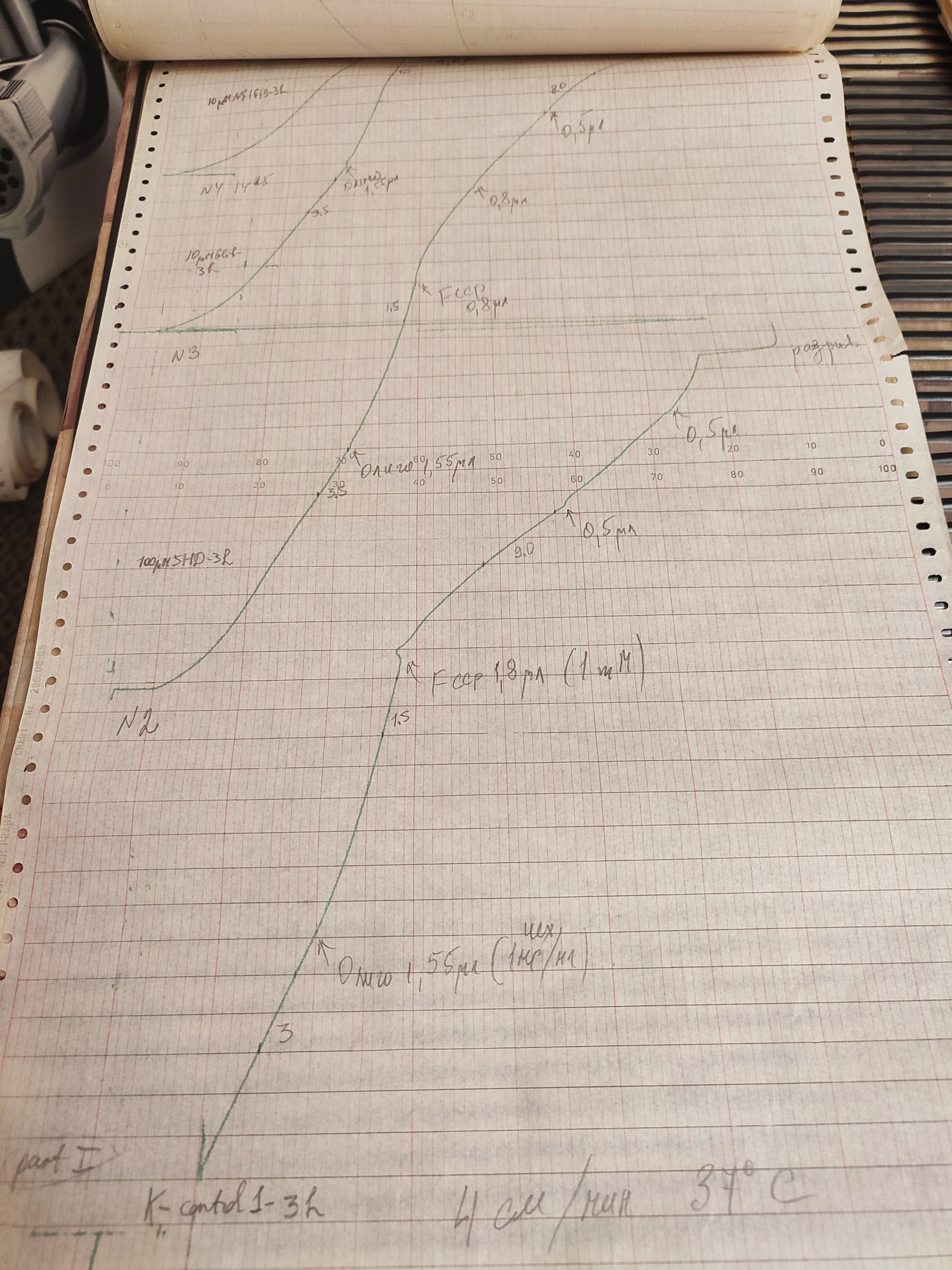

Supplement: Supplementary file 1 [file ijms-26-10048-s001.zip › FigS1-3h-control-PC12cells.jpg]

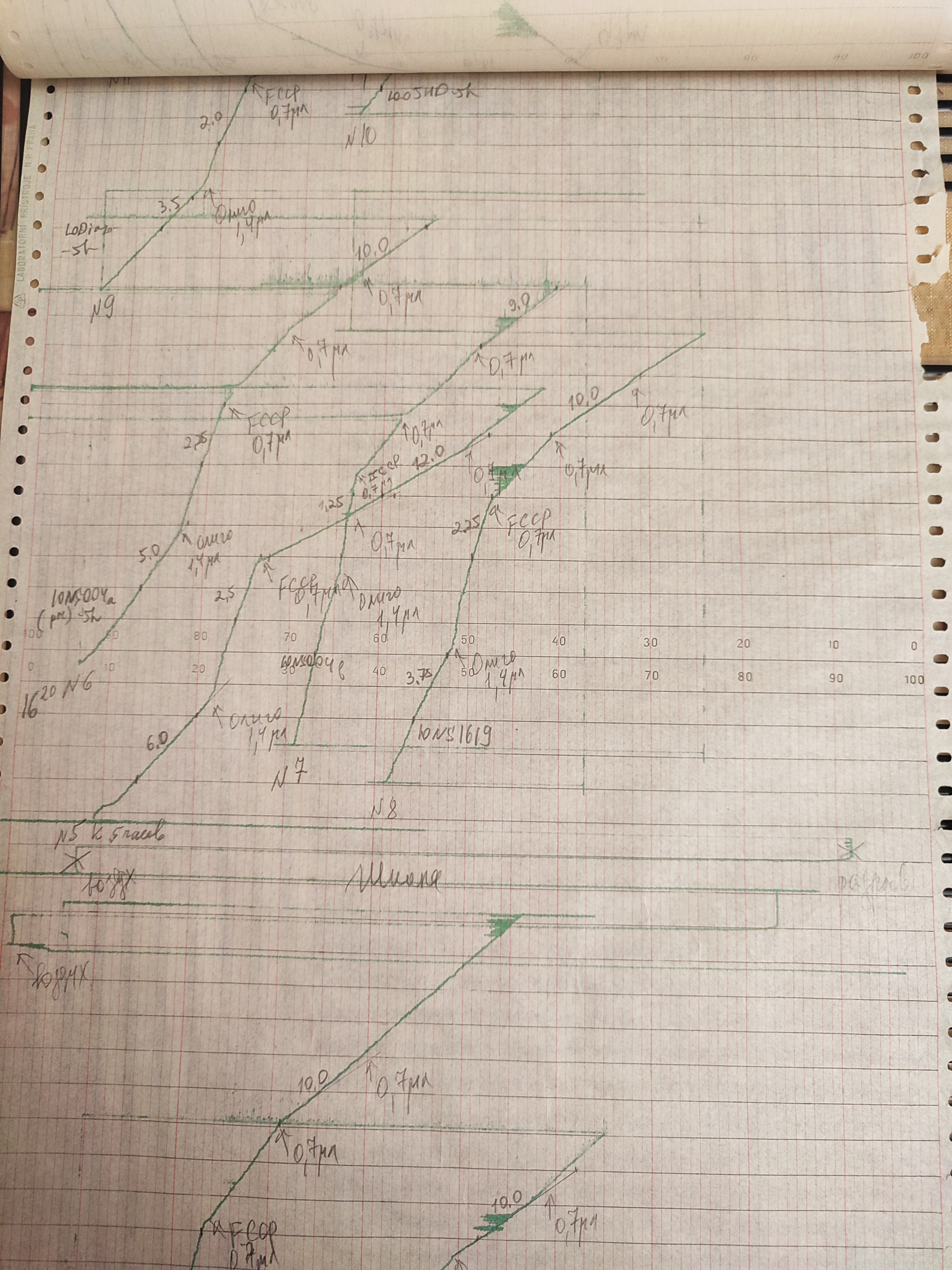

Supplement: Supplementary file 1 [file ijms-26-10048-s001.zip › FigS3-5h-control-10NS004-10NS1619-PC12cells.jpg]

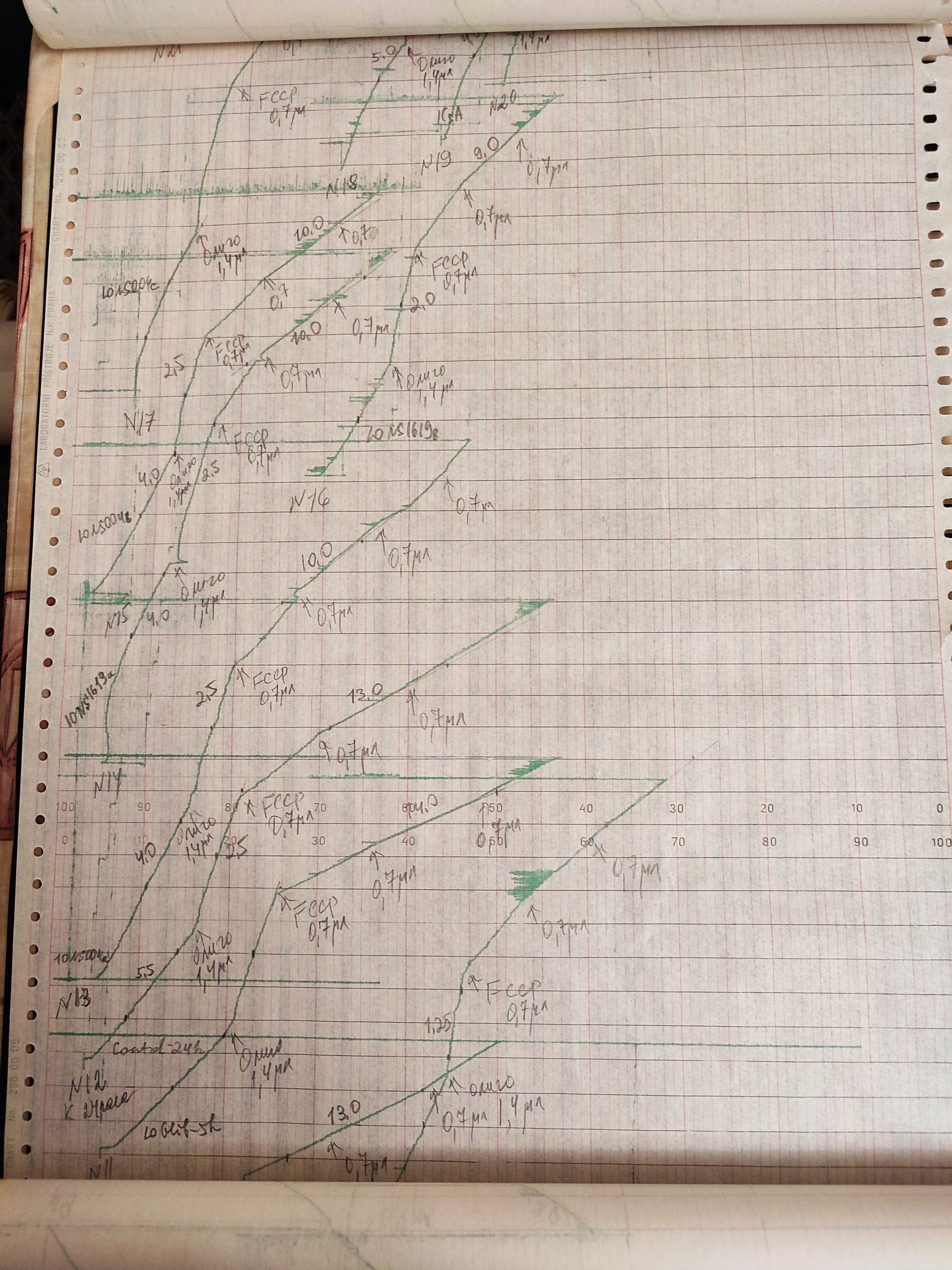

Supplement: Supplementary file 1 [file ijms-26-10048-s001.zip › FigS4-24h-control-10NS004-10NS1619-PC12cells.jpg]
